# Defective pollen meiosis in Arabidopsis due to combined arabinan and galactan insufficiency

**DOI:** 10.1093/pcp/pcaf085

**Published:** 2025-07-25

**Authors:** Takuma Kikuchi, Kouichi Soga, Toshihisa Kotake, Daisuke Takahashi

**Affiliations:** Graduate School of Science and Engineering, Saitama University, 255 Shimo-Okubo, Sakura-ku, Saitama City, Saitama 338-8570, Japan; Graduate School of Science, Osaka Metropolitan University, 3-3-138 Sugimoto, Sumiyoshi-ku, Osaka City, Osaka 558-8585, Japan; Graduate School of Science and Engineering, Saitama University, 255 Shimo-Okubo, Sakura-ku, Saitama City, Saitama 338-8570, Japan; Graduate School of Science and Engineering, Saitama University, 255 Shimo-Okubo, Sakura-ku, Saitama City, Saitama 338-8570, Japan

**Keywords:** arabinan, galactan, male sterility, pectin, pollen development

## Abstract

The development of pollen is critical for seed plants and depends on precise cellular and molecular mechanisms. It is known that the cell wall plays a key role in the progression of pollen maturation. An earlier phase in pollen development is the meiosis of pollen mother cells (PMCs), a fundamental process for producing viable pollen grains. However, the significance of the cell wall during pre-meiosis processes has remained unclear. Pectin, a major component of the cell wall, accumulates abundantly in pollen. To investigate the significance of the cell wall during pre-meiosis and meiosis, we generated an *arad1 gals2 gals3* triple mutant of *Arabidopsis thaliana*, lacking the genes responsible for normal synthesis of arabinan and galactan, which constitute the side chains of pectin. Although vegetative growth and cell wall properties were comparable in wild-type (WT) and *arad1 gals2 gals3*, the pollen development in the mutant failed during meiosis. Immunohistochemical analysis showed that pectic arabinan and galactan accumulated in WT PMCs before meiosis, but this was not observed in mutant PMCs. On the other hand, it was found that pistil development of WT and *arad1 gals2 gals3* was comparable. These findings suggest that the transient accumulation of arabinan and galactan in PMCs before meiosis is crucial for pollen meiosis. We believe that this study is the first to demonstrate the critical role of cell wall components, specifically pectic arabinan and galactan, in the pre-meiosis processes of pollen development.

## Introduction

The development of pollen is a pivotal event for reproduction in seed plants. Pollen development occurs in the anthers and is divided into 14 stages (Sanders et al. 1999, Scott et al. 2004, Alvarez-Buylla et al. 2010). In stage 1, the stamen primordium consists of a uniform tissue surrounded by the epidermis. In stage 2, four anther locules begin to form as archesporial cells emerge, which will give rise to pollen mother cells (PMCs). During stages 3 and 4, the anther locules continue to develop, and the archesporial cells undergo division. In stage 5, the divided archesporial cells differentiate into PMCs, which serve as precursors to pollen grains. In stage 6, the PMCs undergo meiosis, initiating the subsequent progression of pollen development (Sanders et al. 1999). Several cell wall components have been reported to play distinct and important roles in pollen development across various plant species, including Arabidopsis, rice, and tobacco (Worrall et al. 1992, Enns et al. 2005, Blackmore et al. 2007, Ariizumi and Toriyama 2011, Wan et al. 2011, Mollet et al. 2013, Cankar et al. 2014, Shi et al. 2015, Yin et al. 2022, Hasegawa et al. 2023) (Supplementary Table S1). Homogalacturonan (HG) and arabinan are crucial for pollen development and pollen maturation after meiosis in both Arabidopsis and rice, affecting the morphology of the pollen wall (Cankar et al. 2014, Hasegawa et al. 2023). Hasegawa et al. (2023) further proposed that in rice, pectin accumulation in the primary cell wall during early PMC development serves as a scaffold for the subsequent deposition of callose between the plasma membrane and the primary wall. Callose accumulates in the pollen cell wall during and after meiosis, and is essential for the proper morphological development of mature pollen (Worrall et al. 1992, Enns et al. 2005, Wan et al. 2011). Sporopollenin is formed in the outermost layer of the pollen cell wall and, due to its highly durable nature, is suggested to play a crucial role in maintaining pollen morphology and facilitating pollination (Ariizumi and Toriyama 2011).

The cell wall provides mechanical strength, defines cell shape, and influences overall cellular development. Cell wall composition varies across plant organs, imparting distinct characteristics to each. In dicotyledons, the primary cell wall is mainly composed of three polysaccharides: pectin, hemicellulose and cellulose. Pectin and hemicellulose, known as matrix polysaccharides, serve as reinforcers that fill in and connect the cellulose microfibril network (Carpita and Gibeaut 1993), and contribute to the mechanical properties and porosity of the cell wall (Fleischer et al. 1999, Peaucelle et al. 2011, Klaassen and Trindade 2020). Pectin consists of three main domains: HG, rhamnogalacturonan-I (RG-I), and RG-II. HG is a linear polymer of d-galacturonic acid (GalA) that contributes to cell wall integrity via calcium cross-linking, which depends on the degree of HG methylation (Tibbits et al. 1998, Hepler and Winship 2010). RG-II forms a pectin network by cross-linking with boron (Matoh 1997, Fleischer et al. 1999). RG-I is composed of a main chain having repeating sequences of GalA and d-rhamnose (Rha), with side chains of linear β-1,4-galactan composed of d-galactose (Gal), branched α-1,5-arabinan composed of l-arabinose (Ara), and type-II arabinogalactan composed of both Gal and Ara attached to the Rha residue (Kaczmarska et al. 2022). Pectic arabinan, in particular, is involved in mechanisms such as stomatal opening and closing, cell wall flexibility in inflorescence stems, and pollen tube elongation (Jones et al. 2003, Verhertbruggen et al. 2013, Lampugnani et al. 2016, Carroll et al. 2022). Pectic galactan, by contrast, has been proposed to fulfill multiple roles in non-elongating cells, including interactions with cellulose and other matrix polysaccharides, modulation of cell wall mechanical properties, regulation of wall hydrophilicity, and involvement in tolerance to salt and freezing stresses (McCartney et al. 2000, Torode et al. 2018, Klaassen and Trindade 2020, Moneo-Sánchez et al. 2020, Wang et al. 2021a, Gao et al. 2023, Takahashi et al. 2024).

During pollen development and maturation, cell wall composition and structure differ from what is seen during vegetative growth. The pollen wall is composed of two distinct layers called exine (the outer layer) and intine (the inner layer). The exine, which is rich in sporopollenin, begins to accumulate on the microspore surface shortly after meiosis, around stages 6–7, and is largely deposited by the tapetum. The intine is formed later, beginning around stage 11, and consists primarily of pectin, cellulose, and hemicellulose synthesized by the microspore itself (Ma et al. 2021). Furthermore, callose, a linear β-1,3-glucan, is a major component of the pollen cell wall during maturation, and its eventual degradation and utilization is an essential factor in the final maturation of pollen (Dong et al. 2005, Chen and Kim 2009). Callose is a specialized cell wall polysaccharide that accumulates not only in the cell walls of plant pollen but also during specific biological processes such as pathogen defense responses, root hair plug formation, wound responses, the development of sieve plate-like structures in the phloem, and the regulation or closure of plasmodesmata (Currier 1957, Jacobs et al. 2003, Nakashima et al. 2003, Galway et al. 2011, Luna et al. 2011, Xie et al. 2011, Ellinger et al. 2013, Wu et al. 2018, Wang et al. 2021b, Drs et al. 2025). If callose degradation is disrupted, pollen grains develop abnormal morphology, as seen in *Oryza sativa* and *Nicotiana tabacum* (Worrall et al. 1992, Chen and Kim 2009, Wan et al. 2011). This indicates that cell wall components like callose play essential physiological roles in the post-meiotic pollen maturation process.

Pectin, along with callose, has also been recognized as a crucial component of the cell wall involved in pollen development and maturation (Kandasamy et al. 1994, Bárány et al. 2010). Recent studies have highlighted that the degree of methylesterification of HG is essential for both pollen development and maturation (Hasegawa et al. 2023). Pectic arabinan side chains are localized in pollen cell walls and play a significant role in pollen maturation, especially in potato (Dardelle et al. 2010,Castro et al. 2013 , Cankar et al. 2014). Similarly, galactan accumulates in the cell walls of mature pollen in olive and potato, but the expression of enzymes that degrade galactan, as well as the subsequent degradation of galactan, do not appear to affect the morphology or fertilization ability of mature pollen (Castro et al. 2013, Cankar et al. 2014). These observations suggest that galactan plays a less prominent role during the final stages of pollen maturation. Pectic arabinan and galactan are present as neutral sugar side chains in the RG-I region of pectin and may therefore have similar functions in certain physiological processes. However, their specific roles in pollen development and maturation remain largely unknown.

We thus decided to investigate the roles of pectic arabinan and galactan in pollen development in the model plant Arabidopsis. Arabinan is synthesized by two enzymes: ARABINAN DEFICIENT 1 (ARAD1) and ARAD2 (Harholt et al. 2006, 2012). To date, no biochemical activity has been demonstrated for either ARAD1 or ARAD2, and the proposed function of ARAD1 in arabinan biosynthesis remains based solely on phenotypic analyses of mutant plants (Harholt et al. 2006, 2012, Carroll et al. 2022). For ARAD2, functional evidence is even more limited, with no clear phenotypic data available to support a role in arabinan synthesis. Galactan synthesis in Arabidopsis is carried out by three enzymes: GALACTAN SYNTHASE 1 (GALS1), GALS2, and GALS3 (Liwanag et al. 2012, Ebert et al. 2018). Despite the low level of arabinan in *arad1* and *arad2*, and the low level of galactan in *gals1 gals2 gals3*, all these mutants still produce seeds comparable to wild-type (WT). Therefore, the roles of pectin, and particularly arabinan and galactan, in Arabidopsis pollen development have so far remained unclear.

To further examine these roles, we generated an *arad1 gals2 gals3* with reduced levels of both arabinan and galactan in pectin side chains and observed its pollen development. Our results demonstrated that simultaneous reduction of pectic arabinan and galactan causes complete male sterility due to abnormal pollen development. Detailed observations of the pollen development revealed that the polysaccharides in question accumulate on the PMCs surface just before meiosis, and that abnormal morphology of PMCs manifests during meiosis in the mutants. Although several studies have previously shown that cell wall polysaccharides are involved in pollen maturation, this study focuses on their importance in the early stages of pollen development before meiosis.

## Results

### The reduction of arabinan and galactan affects seed formation

**Figure 1:**
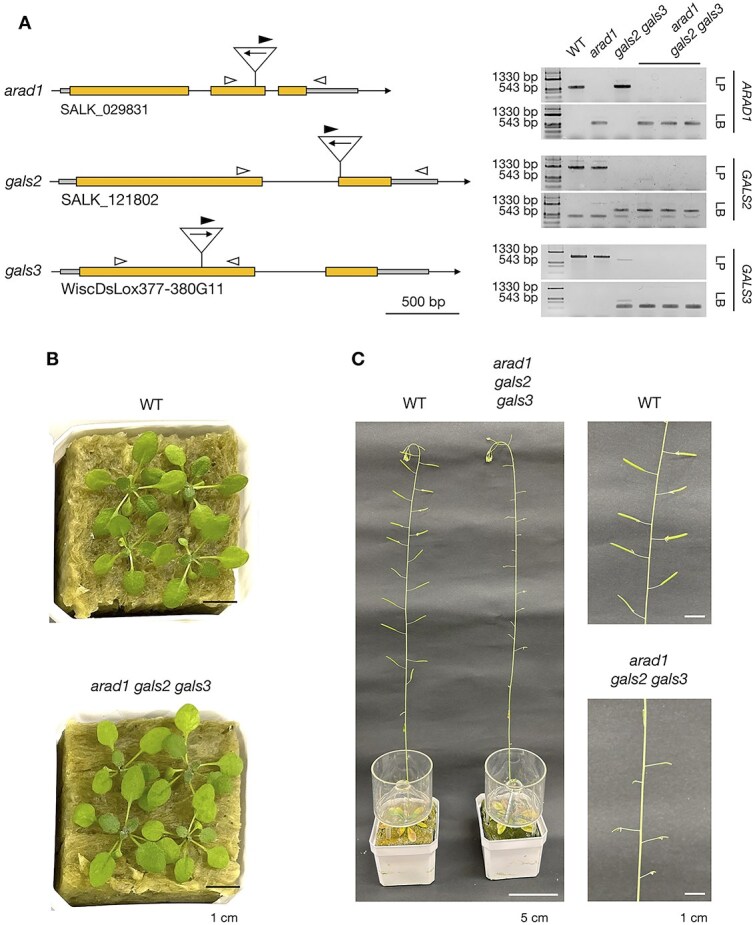
Verification of T-DNA insertion in *ARAD1*, *GALS2* and *GALS3* genes and phenotypes of *arad1 gals2 gals3*. (A) The T-DNA insertion sites for *ARAD1*, *GALS2* and *GALS3* are indicated by arrows, where the direction of the arrow represents the orientation of T-DNA insertion. The arrowheads mark the binding sites of the primers used in this study. White arrowheads indicate gene-specific primers (forward and reverse), and black arrowheads indicate T-DNA border primers used for confirming T-DNA insertions. Scale bar = 500 bp. The figure on the right shows the results of PCR, indicating that T-DNA insertion has occurred in the genes corresponding to each mutant. LP indicates PCR performed with the left (LP) and right (RP) gene-specific primers, while LB indicates PCR performed with the T-DNA-specific left border primer (LB) and RP. (B) Phenotypic observation of rosette leaves in WT and *arad1 gals2 gals3* of seedlings for 3 weeks. Scale bar = 1 cm (C) phenotypic observation of inflorescence stem and siliques in WT and *arad1 gals2 gals3* of seedlings after more than 7 weeks. Scale bar = 5 cm and 1 cm.

We generated an *arad1/+ gals1/+ gals2/+ gals3/+* heterozygous mutant by crossing *arad1* with *gals1 gals2 gals3*. Subsequently, we selected for plants having homozygous T-DNA insertions in three genes using PCR from seeds obtained by self-pollination of heterozygous mutants, yielding seedlings of *arad1 GALS1 gals2 gals3* triple mutant (*arad1 gals2 gals3*, Fig.  1A). As the chromosomal positions of *ARAD1* and *GALS1* are very close to each other (14793567–14795701 bp and 14216771–14219252 bp regions on chromosome 2, respectively), *arad1* and *GALS1* are genetically linked and the obtained *arad1 gals2 gals3* did not have a mutated *GALS1* gene. First, we investigated the influence of *arad1*, *gals2* and *gals3* mutations on the cell wall polysaccharides in the leaves of Arabidopsis. The amount of pectin in the leaves of WT and *arad1 gals2 gals3* was determined by measuring the total sugar content in the EDTA fraction, and the pectin content was found comparable between WT and *arad1 gals2 gals3* (Supplementary Fig. S1A). However, the Ara and Gal of pectin were significantly reduced, with respective decreases of 30% and 25% in mol% composition, and 31% and 27% in sugar content compared to WT (Supplementary Fig. S1B-C). The decrease in Ara and Gal in the EDTA fraction suggested a reduction in arabinan and galactan. On the other hand, in *arad1 gals2 gals3*, there was a tendency for Rha and GalA to increase compared to WT, so that the total pectin content did not change (Supplementary Fig. S1). To see if these components affected the mechanical properties of the cell wall, extensibility and breaking force of the cell wall in leaves were also measured, but *arad1 gals2 gals3* as well as *arad1* and *gals2 gals3* were not significantly different from the WT (Supplementary Fig. S2). Indeed, the reduction of Ara and Gal in the pectin did not affect plant morphology at the vegetative growth stages (Fig.  1B). However, extremely small siliques were observed in *arad1 gals2 gals3* compared to WT (Fig.  1C), likely due to the reduced levels of arabinan and galactan in reproductive tissues.

To examine whether these phenotypes were linked to genotype, seeds produced by self-pollinating *arad1/+ gals1/+ gals2 gals3* heterozygous plants were grown. Subsequently, the genotype of each plant in the next generation was determined by PCR and the number of seeds per silique was counted (Supplementary Fig. S3). The results showed that all *arad1 gals2 gals3* plants were seedless, while *arad1/+ gals1/+ gals2 gals3* had a slightly lower average seed count compared to *gals1 gals2 gals3*. This suggests that both pectic arabinan and galactan are involved in gametogenesis, fertilization, and/or seed formation processes, with arabinan having a greater influence on seed formation than galactan.

### Arabinan and galactan are involved in pollen formation

**Figure 2:**
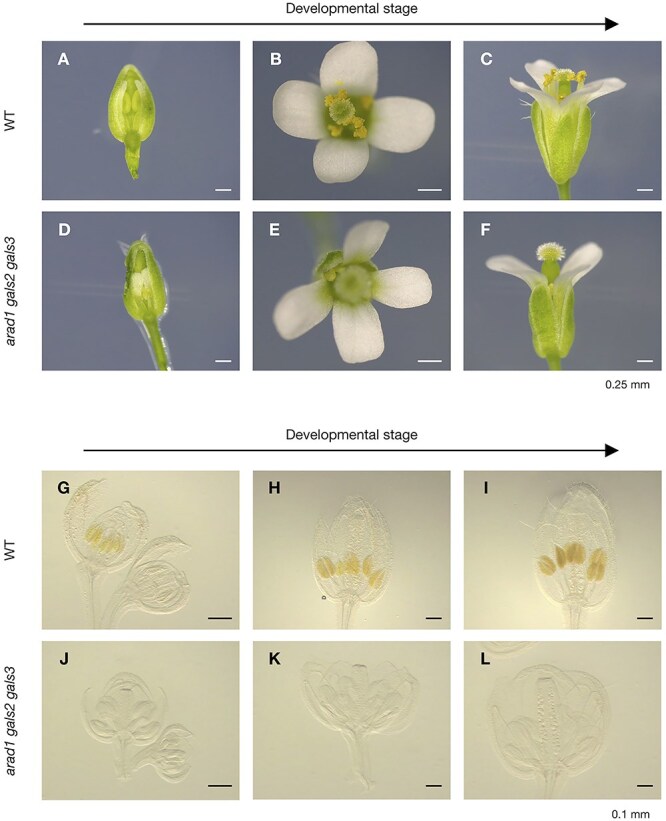
Pollen formation process during the developmental stages of the flower bud observed by optical microscope. (A-C) Development of flower bud morphology in WT. Scale bar = 0.25 mm. (D-F) Development of flower bud morphology in *arad1 gals2 gals3*. Scale bar = 0.25 mm. (G-I) Pollen formation process during the development of flower buds in WT before flowering. Scale bar = 0.1 mm. (J-L) Pollen formation process during the development of flower buds in *arad1 gals2 gals3* before flowering. Scale bar = 0.1 mm.

We therefore tried to determine the reason for the inability of *arad1 gals2 gals3* to produce seed and investigated anthers and pistils under a stereomicroscope. In contrast to WT (Fig. 2A-C), anthers were very short and small in *arad1 gals2 gals3* even though bud development progressed (Fig. 2D-F). Therefore, to examine the formation of anthers in the early stages of floral development, the bud tissue was made transparent, and internal structures were observed with a stereomicroscope. Yellow parts derived from exine, a specialized cell wall structure formed on the pollen surface, was observed in WT, *gals1 gals2 gals3*, *arad1* and *arad2* anthers, whereas no such structures were observed in *arad1 gals2 gals3* (Fig. 2G-L, Supplementary Fig. S4). On the other hand, pistils were normal morphology in *arad1 gals2 gals3* as well as in WT, indicating that the *arad1 gals2 gals3* mutation affects pollen development in the anthers, causing male sterility but does not impinge on pistil development. Indeed, when pollen in WT was transferred to *arad1 gals2 gals3* pistils, the pistils successfully formed seeds and PCR confirmed that they had inherited the genes from the WT (Supplementary Fig. S5).

To determine whether pollen formation in *arad1 gals2 gals3* is normal, F1 seeds resulting from pollination of WT pistils with pollen from *arad1/+ gals1/+ gals2 gals3* were sown on medium, and their genotypes were analyzed (Table 1). The pollen genotypes of *arad1/+ gals1/+ gals2 gals3* at the post-meiotic stage were theoretically either *ARAD1 gals1 gals2 gals3* or *arad1 GALS1 gals2 gals3*. The genotypes of the F1 seeds obtained in this experiment showed a nearly one-to-one ratio of *arad1*/+ *GALS1 gals2 gals3* and *ARAD1 gals1*/*+ gals2 gals3* (Table 1). A chi-squared test confirmed that the observed genotype segregation did not deviate from the expected ratio (Table 1). These results indicated that pollen with *arad1 gals2 gals3* genotype after meiosis has the ability to fertilize normally. Therefore, while arabinan and galactan are essential for seed formation, our results suggest that even when their levels are reduced after meiosis, pollen is still able to retain fertilization capability.

**Table 1:** The relationship between the genotype of pollen after meiosis and male sterility. Pollen collected from plants with *arad1/+ gals1/+ gals2 gals3* genotype was used to pollinate the stigma of WT plants. The genotypes of the resulting F1 population were determined by PCR to identify the pollen genotype from which each seed originated. The differences between the expected and observed segregation ratios of F1 population were statistically analyzed using a chi-squared test.

After meiosis pollen genotype	*arad1 gals2 gals3*	*gals1 gals2 gals3*
Observed	33	38
Expected	0.5	0.5
X-squared	0.3521	
*p*-value	0.5529	

### Arabinan and galactan play an important role in the developmental process of PMCs

**Figure 3:**
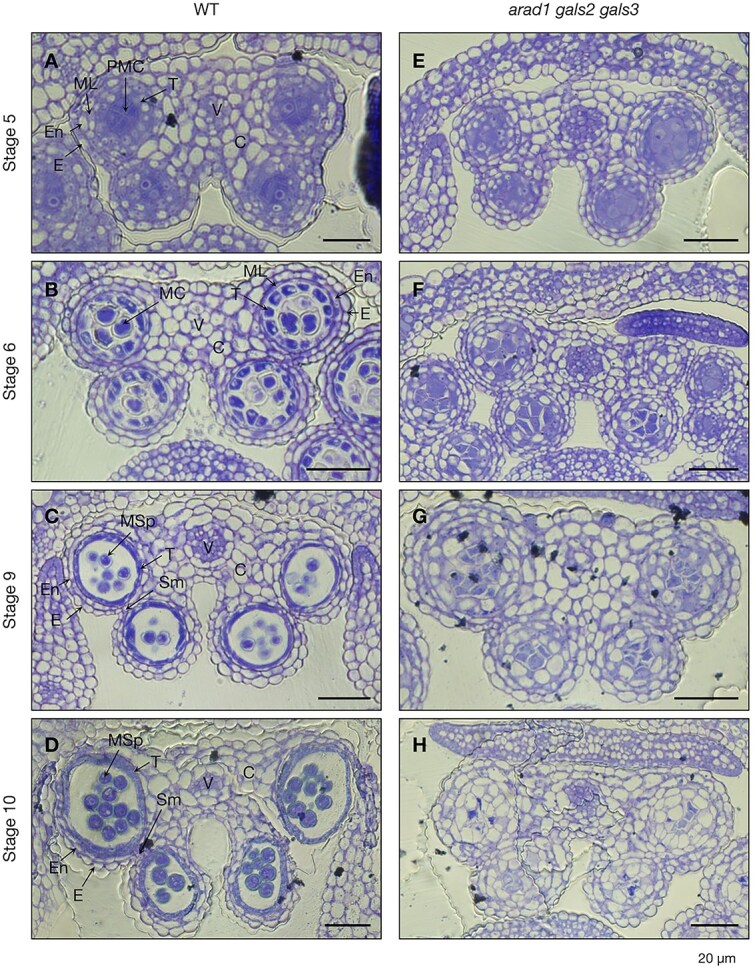
Observation of pollen formation process in WT and *arad1 gals2 gals3* using toluidine blue staining. (A-D) The developmental process of pollen in the WT was observed with toluidine blue staining under an optical microscope, focusing on the progression from meiotic PMCs to microspores. Scale bar = 20 μm. (E-H) The developmental process of pollen in *arad1 gals2 gals3* was observed at various anther developmental stages. Scale bar = 20 μm. C, connective; E, epidermis; En, endothecium; MC, meiotic cell; ML, middle layer; MSp, microspore; PMC, pollen mother cell; SM, septum; T, tapetum; V, vascular region.

To identify developmental defects during the early stage of pollen formation in *arad1 gals2 gals3*, we prepared resin-embedded bud sections and stained the cell walls with toluidine blue (Fig.  3). In WT, pollen development proceeded synchronously with the developmental stages of anthers, with PMCs and microspores were observed at stage 5 to stage 10 (Fig.  3A-D). Similarly normal PMCs and tapetum formation in anthers were observed in *arad1 gals2 gals3* as well as WT at stage 5 (Fig.  3E). However, in *arad1 gals2 gals3*, tapetums were absent in anthers, and no pollen development to stage 6 or beyond was observed in any individuals examined (Fig.  3F-3H). Instead, a different anther internal structure was observed in *arad1 gals2 gals3* than in the WT (Fig.  3F-H), and the PMCs-like structures appeared to have eventually shrunk (Fig.  3H). This suggests that the accumulation of pectic arabinan and galactan around stage 5 is necessary for progression to subsequent stages, such as during PMCs formation and/or the pollen tetrad stage when meiosis is taking place. The observations in the toluidine blue stained sections supported our hypothesis that ARAD1, GALS2 and GALS3 play a role in the pre-meiotic stages of pollen development, but not in the post-meiotic stages or during seed formation.

**Figure 4:**
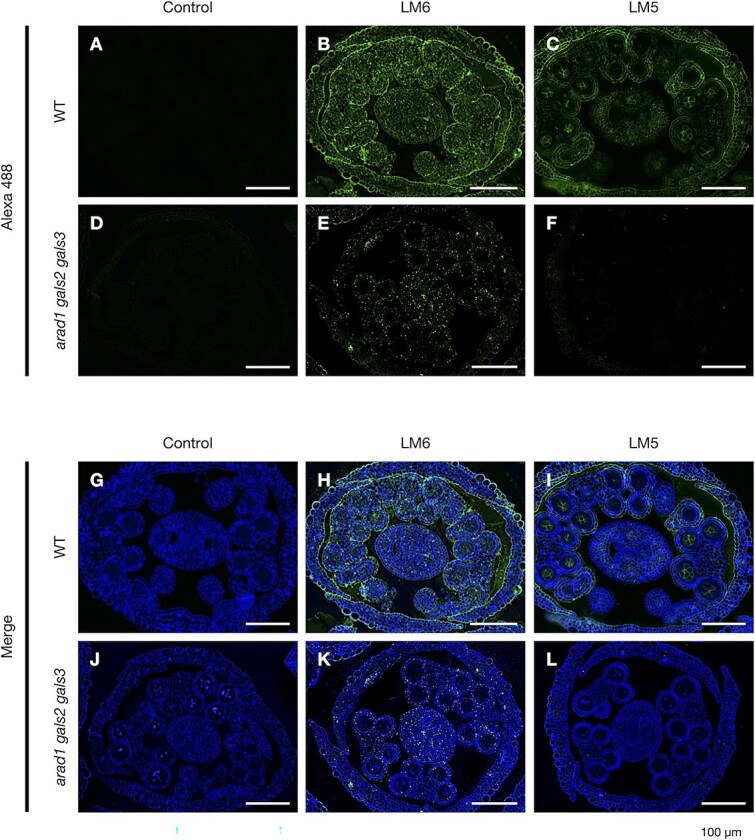
Accumulation of arabinan and galactan in the WT and *arad1 gals2 gals3* anther at stage 5. Observation of arabinan and galactan distribution in cross sections of an entire flower bud during anther and pollen development at stage 5 in WT and *arad1 gals2 gals3*. Scale bar = 100 μm. Control samples were prepared without using primary antibodies such as LM6 or LM5; only Alexa Fluor 488 and calcofluor white were used (A, D, G, J). Green-stained regions indicate arabinan and galactan accumulation detected with LM6 and LM5 respectively, and Alexa fluor 488 antibodies (B, C, E, F, H, I, K, L), and blue-stained regions indicate call wall accumulation with calcofluor white (H, I, K, L).

**Figure 5:**
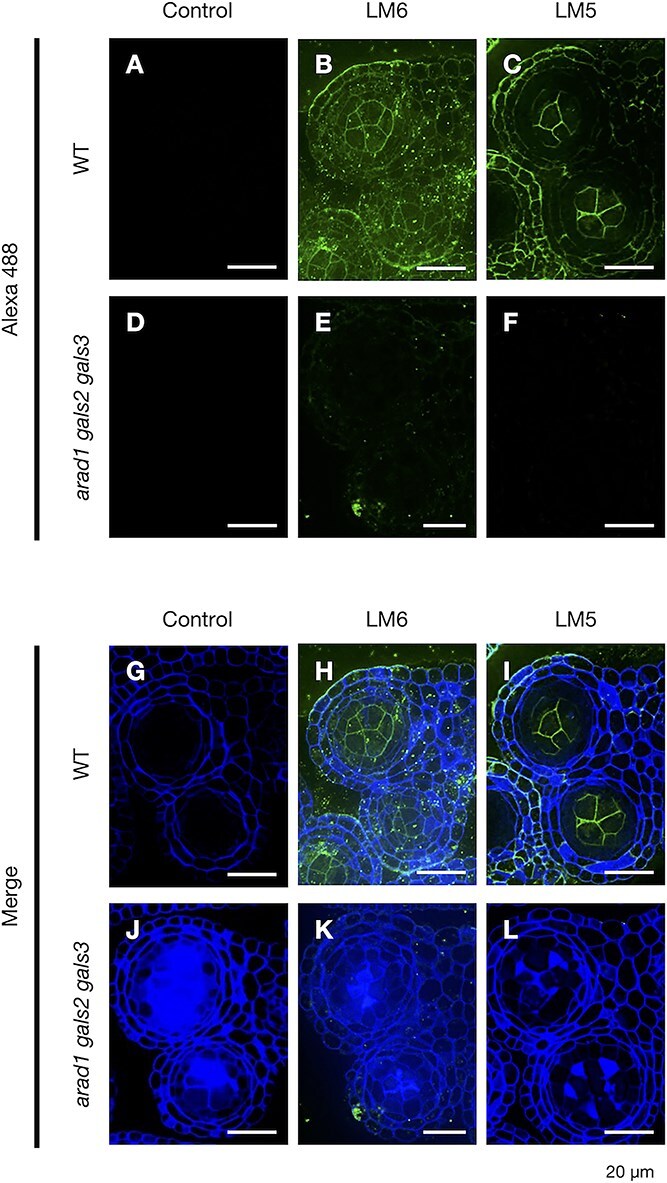
Accumulation of arabinan and galactan in WT and *arad1 gals2 gals3* PMCs at stage5. Zoom in images of arabinan and galactan distribution in cross sections of PMCs at stage 5 in WT and *arad1 gals2 gals3*. Scale bar = 20 μm. Control samples were prepared without using primary antibodies such as LM6 or LM5; only Alexa Fluor 488 and calcofluor white were used (A, D, G, J). Green-stained regions indicate arabinan and galactan accumulation detected with LM6 and LM5 respectively, and Alexa fluor 488 antibodies (B, C, E, F, H, I, K, L), and blue-stained regions indicate call wall accumulation with calcofluor white (H, I, K, L).

**Figure 6:**
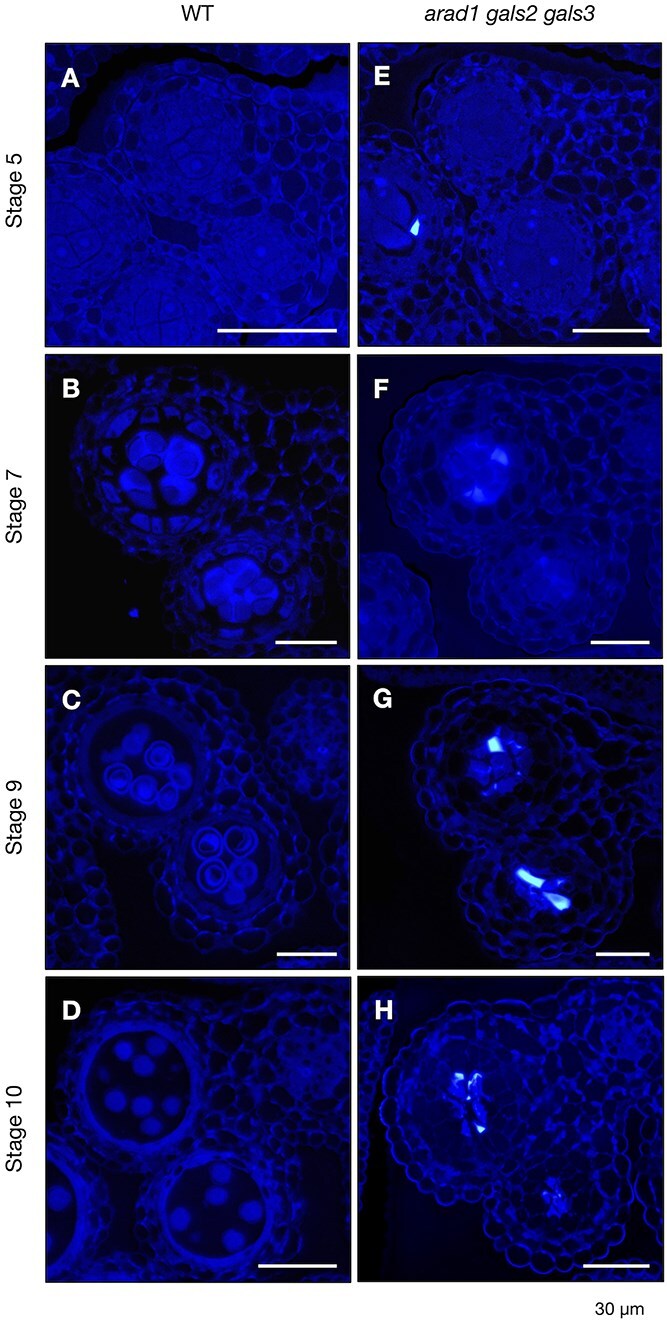
Accumulation of callose during the pollen development process in WT and *arad1 gals2 gals3*. (A-H) The accumulation of callose during the pollen development progress was observed using aniline blue staining in WT (A-D) and *arad1 gals2 gals3* (E-H). Scale bar = 30 μm.

In order to clarify the localization of pectic arabinan and galactan during pollen and anther development, immunohistochemical staining was performed (Figs. 4, 5) (Supplementary Figs. S6, 7). Using LM6 and LM5, antibodies specific to arabinan and galactan, respectively, the localization patterns of these polysaccharides were examined at stages 4, 5 and 10 in both WT and *arad1 gals2 gals3*. At stage 4 in WT, arabinan and galactan were found to accumulate throughout the cell walls of anther tissues (Supplementary Fig. S6A-C, G-I). At stage 5, similar accumulation was observed throughout the anther locules, with particularly strong signals at the surface of the PMCs (Fig. 4A-C, G-I, 5A-C, G-I). In contrast, galactan accumulation in the tapetal tissue was no longer detected in stage 5 (Figs. 4B, H). By stage 10, arabinan and galactan remained in the anther locules but were scarcely detected in the microspores (Supplementary Fig. S7A-C, G-I). In the anthers of *arad1 gals2 gals3*, galactan accumulation was not observed, whereas arabinan accumulation was detected. However, on the PMC, neither arabinan nor galactan accumulation was observed (Fig. 4D-F, J-L, 5D-F, J-L) (Supplementary Fig. S6D-F, J-L, S7D-F, J-L). In addition, arabinan accumulation was observed in the anthers and on the PMCs in both *arad1* and *arad2* (Supplementary Fig. S8). These findings suggest that the transient accumulation of arabinan and galactan during specific stages of pollen development is critical for proper pollen maturation and galactan may be required for arabinan accumulation on the PMCs. Additionally, a thick cell wall structure that stained strongly with calcofluor white was observed in *arad1 gals2 gals3* (Fig. 4J-L, 5J-L). This structure was also stained with aniline blue, indicating the presence of callose (Fig.  6). Callose accumulation in PMCs was especially prominent during meiosis and was similarly observed in both genotypes. However, in *arad1 gals2 gals3*, callose continued to accumulate even after the PMCs failed to develop normally and collapsed (Fig.  6G, H).

Additionally, since LM6 is known to recognize not only arabinan but also the proteoglycan arabinogalactan protein (AGP), we examined whether the LM6 staining signals might be derived from AGP by using LM2, an antibody that specifically recognizes AGP (Verhertbruggen et al. 2009). Immunohistochemical staining with LM2 showed comparable levels of fluorescence in both the WT and the *arad1 gals2 gals3* (Supplementary Fig. S9A-B, E-F, I-J, M-N). Moreover, LM2 staining suggested that AGPs do not accumulate in the cell walls of PMCs. By contrast, staining with LM6, which detects arabinan, revealed reduced fluorescence in the PMC cell walls of *arad1 gals2 gals3* compared to WT (Fig. 5B, E, H, K). These findings strongly suggest that arabinan plays a crucial role in PMC development. In addition, we investigated the accumulation of other cell wall components known to affect pollen development, specifically high- and low-methylesterified HG, using LM20 and LM19, respectively. The results showed that, at stage 5, neither form of HG accumulated in the cell walls of PMCs or anther tissues (Supplementary Fig. S9C-D, G-H, K-L, O-P), suggesting that these components are unlikely to contribute to the phenotypes observed in *arad1 gals2 gals3*. Together, these results indicate that HG is either not substantially accumulated or not accessible in the PMC walls at this stage, and that methylesterification status does not explain the mutant phenotype.

## Discussion

This study demonstrates that arabinan and galactan are crucial for normal pollen development. Previous research has indicated that significant reductions of arabinan in *arad1* or galactan in *gals1 gals2 gals3* did not notably affect pollen development or plant growth (Harholt et al. 2006, 2012, Liwanag et al. 2012). In addition, a previous study using transgenic potatoes overexpressing arabinan- and galactan-degrading enzymes showed that galactan had little effect on pollen formation, whereas arabinan played an important role in the pollen maturation process (Cankar et al. 2014). However, in this study, the simultaneous reduction of arabinan and galactan in *arad1 gals2 gals3* showed minimal effects on growth but resulted in male sterility, preventing seed production. Intriguingly, arabinan accumulation was not observed on the PMC in *arad1 gals2 gals3*, whereas it was retained in *arad1* (Fig. 5E, K; Supplementary Fig. S8B, F). This implies that galactan might affect deposition and/or stability of arabinan in early pollen development. Therefore, we speculate that both arabinan and galactan are required together to maintain proper RG-I structure and its interaction with cellulose and other wall components, which in turn ensures the mechanical integrity or remodeling capacity of the PMC wall during early pollen development. The concurrent loss of both types of side chains in PMC may disrupt this cooperative function, thereby compromising pollen meiosis.

Analysis of mRNA expression retrieved from the eFP browser (https://bar.utoronto.ca/efp/cgi-bin/efpWeb.cgi) and a number of research articles, revealed that *ARAD1*, *GALS2* and *GALS3* are expressed in various tissues, including stamens (Harholt et al. 2006, Liwanag et al. 2012) (Supplementary Fig. S10–12). However, in the *arad1 gals2 gals3* mutant, no phenotypic changes were observed in leaves, stems, or petals and the defects were confined specifically to pollen. By contrast, other male sterile mutants with mutations in transcription factor, such as the *apetala1* (*ap1*), *ap2* and the *leunig* (*lug*) mutants, exhibit additional phenotypes, including floral organ abnormalities and altered leaf morphology (Irish and Sussex 1990, Jofuku et al. 1994, Shi et al. 2024). Nonetheless, these mutants share a similarity with *arad1 gals2 gals3* in that some pollen grains exhibit abnormalities prior to meiosis. Although it remains unclear whether these transcription factors are directly involved in cell wall regulation, they may act upstream of *ARAD1*, *GALS2* and *GALS3*, potentially influencing their expression. Therefore, investigating these transcription factors may provide deeper insight into the relationship between the pollen cell wall and pollen development. These findings highlight the essential roles of arabinan and galactan in seed formation and suggest that the transcriptional regulation of *ARAD1*, *GALS2* and *GALS3* may be critical for proper pollen development.

The inability of *arad1 gals2 gals3* to produce seeds appears to be due to the failure of pollen development (Fig. 2, 3). It is known that cell wall components are involved in pollen maturation (Dong et al. 2005, Enns et al. 2005, Cankar et al. 2014, Hasegawa et al. 2023), particularly pectin, where the methylesterification of HG in rice and arabinan in potato has been shown to be essential for normal pollen formation (Cankar et al. 2014, Hasegawa et al. 2023). However, in Arabidopsis, *arad1*, *arad2* and *gals1 gals2 gals3* can produce pollen similarly to the WT (Supplementary Fig. S4). Thus, the role of arabinan and galactan in pollen development was largely unknown. Our study demonstrates that both arabinan and galactan contribute to early pollen development prior to meiosis in Arabidopsis. However, several lines of evidence suggest that arabinan may play a more dominant role. First, viable seeds are produced in the *gals1 gals2 gals3* triple mutant, which lacks galactan, indicating that galactan is not essential for pollen functionality. Second, Arabidopsis mutants deficient in UDP-l-Ara*f* synthesis due to defects in RGP1 and RGP2, fail to form viable seeds, underscoring the essential role of arabinan (Drakakaki et al. 2006). Third, in potato, transgenic lines expressing arabinan-degrading enzymes show reduced pollen viability, while galactan degradation has little effect (Cankar et al. 2014). Taken together, these findings support the idea that arabinan is more important than galactan for successful pollen and seed development (Supplementary Fig. S3). In addition, this study showed that arabinan accumulation was observed on the PMCs surface in the *arad1*, but not in the *arad1 gals2 gals3* mutant, suggesting that galactan may be involved in arabinan accumulation in PMCs.

Callose is known to be the cell wall component most closely involved in pollen maturation (Dong et al. 2005, Enns et al. 2005, Chen and Kim 2009). In the present study, a certain level of callose accumulation occurred even after a stage in which no accumulation of arabinan and galactan was observed in the *arad1 gals2 gals3* (Fig. 6). These results suggest that arabinan and galactan play key roles during PMCs meiosis, and their joint reduction causes male sterility independently of callose accumulation.

In addition, AGPs and extensins have been suggested to be important for pollen maturation (Stratford et al. 2001, Coimbra et al. 2009, 2010, Wang et al. 2018, Jaffri et al. 2023, Kaur et al. 2023). Specifically, in rice, mutants of Hyp-O-GalTs, which modify AGPs with Gal, exhibit reduced pollen production and abnormalities in pollen tube length, leading to male sterility (Kaur et al. 2023). Furthermore, the loss of FIN4 in tomato, which is responsible for the arabinosylation of extensins, results in abnormalities during the post-meiotic pollen maturation process (Jaffri et al. 2023). In the *fin4* mutant, the exposure of both low-methylesterified and high-methylesterified HG is increased, suggesting that extensins serve as a scaffold for pectin synthesis in pollen (Jaffri et al. 2023). It has also been shown that the amount of HG and its methylesterification are tightly regulated during pollen development in tomato (Jaffri and MacAlister 2021). These studies highlight that AGPs, extensins, and pectic components such as HG play important roles in pollen development, particularly during the post-meiotic stages. However, it is important to note that these studies were conducted in different species and often focused on later stages of pollen maturation. In contrast, our study examined the meiotic stage in Arabidopsis and found that arabinan and galactan are critical for this earlier phase of development. In addition, the lack of LM19 and LM20 signals in PMCs at stage 5 suggests that HG either does not accumulate or is not accessible at this stage in Arabidopsis. Therefore, the reduced staining of LM19 and LM20 observed in our study may reflect differences in species, developmental timing, or epitope accessibility. Together, these considerations support the idea that arabinan and galactan contribute to pollen development via a distinct mechanism, particularly during meiosis.

Arabinan and galactan are neutral sugar side chains of pectin and it has been suggested that they bind to cellulose microfibrils (Lin et al. 2015). This interaction is thought to enhance cell wall strength and create appropriate spacing between pectin main chains including HG (Moore et al. 2008, Verhertbruggen et al. 2009, Harholt et al. 2010, Mariette et al. 2021). In this study, arabinan and galactan were observed to accumulate at the cell adhesion surfaces of PMCs. These components are deposited in the primary cell wall, but they may also accumulate in the middle lamella. The middle lamella, primarily composed of pectin, acts as an adhesive layer between adjacent primary cell walls (Zamil and Geitmann 2017). During the synthesis of HG in the Golgi apparatus, it is methylesterified (Wolf et al. 2009, Wolf and Greiner 2012), but its de-methylesterification, facilitated by pectin methylesterase (PME), plays a critical role in the adhesive properties of the pectin and thus of the middle lamella (Bosch and Hepler 2005, Pelloux et al. 2007). De-methylesterified HG, with nine or more consecutive de-methylated GalA units, can form calcium-mediated cross-links, leading to gelation (Wolf et al. 2009). In Arabidopsis, PME has also been implicated in the separation of PMCs into a tetrad after meiosis (Francis et al. 2006). This separation process requires de-methylesterified HG, which can be cleaved by endo-polygalacturonases (Wolf et al. 2009). The cleavage is effective only once HG has undergone adequate de-methylesterification (Wolf et al. 2009, Sénéchal et al. 2014). Based on previous studies, we hypothesized that the abnormal meiotic phenotype observed in *arad1 gals2 gals3* could be due to altered HG de-methylesterification. However, our immunohistochemical analysis using LM19 and LM20 showed that HG was barely detectable in the PMCs at stage 5 in both WT and mutant plants (Supplementary Fig. S9). This suggests that RG-I side chains may play a more upstream and fundamental role in establishing the appropriate cell wall environment required for meiotic progression, distinct from the known PME-dependent pathways. Further biochemical analyses, such as methylesterification assays or CoMPP, will be necessary to explore this possibility in more detail.

In Arabidopsis, UDP-l-Ara*f* is synthesized from UDP-l-Ara*p* through the mediation of reversibly glycosylated polypeptides (RGPs) (Drakakaki et al. 2006). In rice, this conversion is facilitated by UDP-arabinopyranose mutase (UAM) (Sumiyoshi et al. 2015). Mutants lacking RGPs or UAM have been reported to exhibit abnormal morphology in mature pollen (Drakakaki et al. 2006, Sumiyoshi et al. 2015), underscoring the importance of UDP-l-Ara*f* not only for arabinan biosynthesis, but also for AGP and extensin glycosylation.

Furthermore, arabinan and galactan may influence not only pollen development but also the development of other floral organs. In this study, we also observed that filaments in *arad1 gals2 gals3* appeared to be shorter than in WT (Fig. 2), suggesting that the accumulation of arabinan and galactan may play a significant role in filament development. To date, there have been no reports linking arabinan or galactan accumulation to filament development. Therefore, further analysis focusing on the *arad1 gals2 gals3* mutant and the roles of arabinan and galactan may also provide valuable insights into the relationship between cell wall components and filament development.

In conclusion, this study demonstrates that arabinan and galactan function cooperatively, particularly during pollen meiosis, while having no apparent effect on ovule development, at least in Arabidopsis. Phylogenetic analysis of the ARAD and GALS protein families revealed that ARADs have homologous protein sequences across a wide range of plant species, including lycophytes and angiosperms (Supplementary Fig. S13). Moreover, GALS2 and GALS3 were found to belong to the same clade, whereas GALS1 was placed in a distinct clade (Supplementary Fig. S13). These results suggest that GALS2 and GALS3 in Arabidopsis may have redundant functions, while GALS1 may play a different role. In future studies, it will be essential to elucidate the functions and contributions of arabinan and galactan during the pre-meiotic stage of pollen development in Arabidopsis. Since arabinan and galactan have been observed to accumulate on the surface of PMCs, they may contribute to the mechanical or biochemical properties of PMC walls. However, their precise roles remain unclear. To address this, inducible expression systems for *ARAD1* and *GALS* genes could be developed to control the timing of polysaccharide synthesis. Combined with live-cell imaging and biomechanical assays, such approaches could help uncover how specific cell wall modifications regulate PMC fate and the onset of meiosis. Ultimately, integrating these data could lead to the construction of a spatial–temporal model of cell wall polysaccharide deposition during early anther development, providing a framework for understanding how wall dynamics shape reproductive development in flowering plants.

## Materials and Methods

### Plant materials and growth conditions

WT Arabidopsis Columbia-0 seedlings were sown on Murashige and Skoog agar medium containing 2% sucrose and grown for 1 week at 22°C under a 16-hour light period (120 μmol m^−2^ s^−1^) followed by an 8-hour dark period. Subsequently, they were transplanted into rock wool and grown under the same conditions. The plants used were *arad1* (SALK_029831) (Harholt et al. 2006), lacking *ARAD1*, related to arabinan synthesis, and *gals1 gals2 gals3* (SALK_016687, SALK_121802, WiscDsLox377-380G, respectively) (Ebert et al. 2018), lacking *GALS1*, *GALS2* and *GALS3*, related to galactan synthesis. Stamens and pistils of the *arad1* and *gals1 gals2 gals3* were used for crossing. The resulting seeds were grown and screened by PCR.

### PCR analysis

Approximately half of a plant cotyledon was excised and placed in an Eppendorf tube. 20 μl of PrepMan Ultra (Thermo Fisher Scientific) was added, and the tissue was disrupted. The sample was then heated at 95°C for 20 minutes, followed by the addition of 80 μl of TE buffer (10 mM Tris–HCl, pH 8.0; 1 mM EDTA-2Na). For PCR, 0.5 μl of the extracted genomic DNA and designated primers (Supplementary Table S2) were added to 9.5 μl of PCR mix (EmeraldAmp Max PCR Master Mix; designated primers). The PCR conditions were as follows: 35 cycles of 98°C for 10 seconds, 63°C for 30 seconds, and 72°C for 1 minute, followed by a final extension at 72°C for 7 minutes. The PCR products were stored at 25°C. Subsequently, TAE buffer (Tris; glacial acetic acid; EDTA-2Na) was poured into the electrophoresis tank, and the agarose gel was set in the electrophoresis chamber (ADVANCE, Mupid-exU). Ten μl of each PCR product were loaded into the gel wells and electrophoresed at 100 V for 20 minutes. After electrophoresis, the gel was visualized using a gel documentation system (ATTO, WSE-6100H-CP LuminoGraph I).

### Extraction of the pectic polysaccharides and sugar composition analysis

This method was carried out following the procedure described in previous study (Takahashi et al. 2024). The plant parts except for the roots were ground using a mortar and pestle, then centrifuged at 21,500 × *g* at 4°C for 5 minutes. The supernatant was collected to remove soluble sugars. The precipitates were suspended in 800 μl of 80% ethanol, heated at 100°C for 2 minutes, and centrifuged at 21,500 × *g* at 4°C for 5 minutes. The supernatant was discarded to remove lipids and pigments. Then, 500 μl of water and 500 μl of α-amylase reaction solution (50 mM MOPS-KOH, pH 6.5; α-amylase, 20 units/ml, SIGMA) were added to the precipitate, shaken at 37°C for 2 hours, centrifuged at 21,500 × *g* at 4°C for 5 minutes, and the supernatant was discarded to remove starch. The precipitate, resuspended in 500 μl of water was boiled at 100°C for 10 minutes, centrifuged at 21,500 × *g* at 4°C for 5 minutes, and the supernatant was discarded as part of the hot water fraction. Subsequently, 500 μl of water was added to the precipitate and the suspension was boiled at 100°C for 10 minutes, centrifuged at 21,500 × g at 4°C for 5 minutes, and the supernatant was again discarded as part of the hot water fraction, which was enriched in glucose derived from starch that could not digested by α-amylase and contained trace amounts of low molecular pectin. Then, 500 μl of EDTA solution (50 mM sodium phosphate, pH 6.8; 50 mM EDTA-2Na, pH 8.0) was added to the precipitate and the suspension was boiled at 100°C for 10 minutes, centrifuged at 21,500 × *g* at 4°C for 5 minutes, after which the supernatant was collected as EDTA fraction. Subsequently, 500 μl of EDTA solution was added to the precipitate and after boiling at 100°C for 10 minutes, the suspension was centrifuged at 21,500 × *g* at 4°C for 5 minutes, and the supernatant was collected as the EDTA fraction enriched in high molecular weight pectin. The total pectin content was determined by the phenol-sulfuric acid method (DuBois et al. 1956).

The EDTA fractions obtained by fractionation were dialyzed with water for 2 days and both hot water and EDTA fractions were lyophilized. The extracted cell wall polysaccharides (50 μg) were dissolved in 100 μl of water and 100 μl of 4 N trifluoroacetic acid (TFA) was added to a final concentration of TFA of 2 N. Subsequently, they were hydrolyzed by heating at 120°C for 60 minutes. TFA contained in the sample was removed by drying in a Speed Vac (TAITEC VC-36R, Japan). Then, 50 μl of water was added to the sample, and centrifugation was performed. This process was repeated three times to completely remove the TFA. The 50 μl portion was used for sugar composition analysis by high-performance anion exchange chromatography-pulsed amperometric detection (HPAEC-PAD) with the ICS-5000+ series system equipped with a CarboPac PA-1 column (ThermoFisher Scientific, USA). Elution was carried out with water, 0.1 M sodium hydroxide and 0.1 M sodium hydroxide with 0.5 M sodium acetate at a flow rate of 1.0 ml/min at room temperature (RT) as described previously (Ishikawa et al. 2000).

### Morphological observation of bud interior

Non-flowering buds of WT and *arad1 gals2 gals3* triple grown for more than 50 days were collected. Fixing solution (1.75 M acetic acid in ethanol) was added, vacuum infiltrated in a desiccator and allowed to stand at RT for 2 hours. The fixing solution was removed and subsequently 90% ethanol was added, vacuum infiltrated, and allowed to stand at RT for 20 minutes. These steps were repeated with 70% ethanol, 50% ethanol, 30% ethanol and water. Transparency solution (40 g chloral hydrate in 5 ml glycerol and 10 ml water) was added and allowed to stand overnight at RT. Then, the transparent buds were observed by a stereomicroscope (S9i, Leica Microsystems, Germany).

### Observation of pollen by immunohistochemical staining

Plants were grown for more than 50 days, and non-flowering buds were collected. Fixing solution (10 ml 4% paraformaldehyde; 250 μl 25% glutaraldehyde) was added, the buds were vacuum infiltrated in a desiccator and allowed to stand at RT for 6 hours. The fixing solution was removed and 1 × PBS was added to the samples. Then, samples were vacuum infiltrated and allowed to stand at RT. The same steps were repeated with the following solutions: 10% ethanol (6 hours), 30% ethanol (12 hours), 50% ethanol (12 hours), 70% ethanol (12 hours), 80% ethanol (12 hours), 90% ethanol (12 hours), 100% ethanol (12 hours), and 100% ethanol (12 hours). A mixture of Technovit solution (100 ml Technovit 7100 supplemented with 1 g Hardener I) (Kulzer Technic, Germany) and ethanol (Technovit: ethanol = 1: 5) was added and samples were allowed to stand for 6 hours at 4°C. The same steps were repeated with the following solutions: Technovit: ethanol = 1:3 (12 hours), 3:2 (12 hours), 5:1 (12 hours), 1:0 (12 hours), and 1:0 (12 hours). After Technovit replacement, embedding solution (1 ml Technovit solution supplemented with 100 μl Hardener II) were added and allowed to stand for 12 hours at 4°C. The samples were then incubated at 60°C for 1 hour for curing.

Embedded samples were cut and sectioned using a rotary microtome (Leica, MR2125RT, Germany). Sections were adhered to MAS glass slides (MATSUNAMI, MAS-01, Japan) and used as samples for microscopy. Blocking buffer containing 3% (w/v) skim milk in PBS (0.137 M NaCl; 2.7 mM KCl; 10 mM Na_2_HPO_4_; 1.8 mM KH_2_PO_4_, pH 7.4) was added to the sections on the glass slides and allowed to stand for 30 minutes. The blocking buffer was removed, and 3 μl each of LM5 and LM6 antibodies (diluted 5-fold in blocking buffer, Kerafast, USA), which recognize pectic arabinan and galactan, respectively, were added and allowed to incubate for 2 hours in the dark at RT. The primary antibody was then removed and washed three times with PBS. Subsequently, 3 μl of secondary antibody (Alexa Fluor 488, Thermo Fisher Scientific, USA) was added and allowed to stand for 1 hour in the dark at RT. The secondary antibody solution was then removed and washed three times with PBS. In addition, 3 μl of calcofluor white (2.5 mg calcofluor white in 10 ml PBS) was added and samples were allowed to stand for 10 minutes in the dark at RT. The samples were then washed three times with PBS. The sections were observed by an epifluorescence microscope (ECLIPSE Ci-L Plus, Nikon, Japan).

For toluidine blue and aniline blue staining, sections obtained above were stained with 0.05% toluidine blue (Waldeck GmbH, Germany) in water (1 minute at RT) and 0.01% aniline blue (FUJIFILM Wako Pure Chemical, Japan) in 77 ml phosphate buffer (10 minutes at RT), respectively. The sections were then washed three times with water and observed by an epifluorescence microscope.

### Measurement of mechanical properties of the cell wall

This was carried out following the procedure described in a previous study (Takahashi et al. 2024). For measurement of mechanical properties of the cell wall, the fifth leaf of the plant was harvested and cut into strips of 1 mm width. The strips were immediately immersed in 80% ethanol and allowed to sit for at least 1 week. These samples were subjected to a tensile tester (Tensilon STB-1225S, A&D Co. Ltd., Tokyo, Japan) to determine extensibility and stiffness of the tissue cell wall. Each leaf strip was clamped at 1 mm intervals and stretched at a rate of 20 mm per min until it broke. The cell wall extensibility (strain/load in units of μm/g) was determined by measuring the load rate increase from 4 to 5 g.

### Phylogenetic tree analysis

Amino acid sequences of ARAD and GALS proteins for each plant species were obtained from the Phytozome Database (https://phytozome-next.jgi.doe.gov). The amino acid sequences were aligned using ClustalW and then a phylogenetic tree was created using MEGA11. The distance between each branch was determined using the neighbor-joining method with 1,000 bootstrap samples. The amino acid sequences used in this study are listed in Supplementary Table S3.

### Statistical analysis

Statistical analyses of the data ware based on Tukey-HSD multiple comparison test after the one-way ANOVA or Student's t-test. All calculations were performed with at least three independent biological replicates.

## Supplementary Material

pcp-2025-e-00035-File009_pcaf085

pcp-2025-e-00035-File010_pcaf085

## Data Availability

The data underlying this article are available in the article.
