# Supplementary material for: Defective pollen meiosis in Arabidopsis due to combined arabinan and galactan insufficiency
Source: Plant Cell Physiol. 2025 Jul 25;66(9):1346–59. doi: 10.1093/pcp/pcaf085 (PMC12461841; doi:10.1093/pcp/pcaf085)
Supplement: pcp-2025-e-00035-File009_pcaf085 [file pcp-2025-e-00035-file009_pcaf085.pdf]

Stage	Cell wall component	Species	Reference
Post-meiosis	Homogalacturonan	Arabidopsis	Preuss et al., 1994
Post-meiosis	Homogalacturonan	Rice	Yin et al., 2022
Post-meiosis	Arabinan	Potato	Cankar et al., 2014
Post-meiosis	Callose	Tabacco	Worrall et al., 1992
Post-meiosis	Callose	Arabidopsis	Enns et al., 2005
Post-meiosis	Callose	Rice	Wan et al., 2011
Post-meiosis	Sporopollenin	Rice	Ariizumi and Toriyama, 2011
Post-meiosis	Sporopollenin	Arabidopsis	Ariizumi and Toriyama, 2011
Pre-meiosis?	Homogalacturonan	Rice	Hasegawa et al., 2023
**Pre-meiosis**	**Arabinan and galactan**	**Arabidopsis**	**This study**

Table S1. Past research on the relationship between pollen development and cell wall components.

AGI	Gene name	Mutant accession	Genotyping primers (5'-3')<br>Left primer	T-DNA border primer	Right primer
At2g35100	*ARAD1*	SALK_029831	CCAGAGAGAATCGACGAGC	ATTTTGCCGATTTCGGAAC	TCAAGCTCCTCCACAGTCC
At5g44670	*GALS2*	SALK_121802	CGACAAAGATCCATGGAAGAC	ATTTTGCCGATTTCGGAAC	TCGGTGATGGTATCTTTGGAG
At4g20170	*GALS3*	WiscDsLox377-380G	TCCGATTTCCGCTTCTGCAT	AACGTCCGCAATGTGTTATTA	ACGAAATCGTCTCTTTCACTGG

Table S2. Primer sequences used in this study.

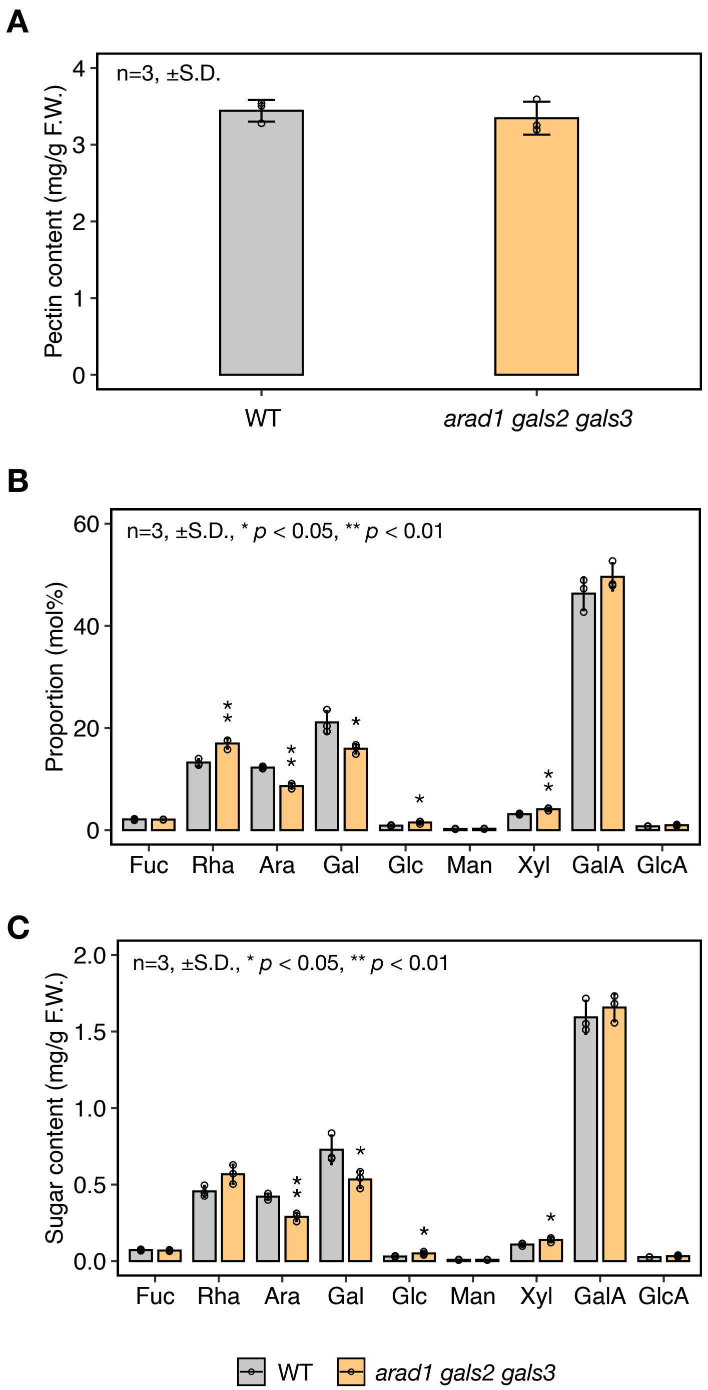

Fig. S1. Pectin content, monosaccharide composition ratio, and monosaccharide content in the aboveground parts of WT and *arad1 gals2 gals3*. (A) The pectin content (EDTA fraction) of three-week-old seedlings of WT and *arad1 gals2 gals3* was measured. Values are means ± S.D., n = 3. (B) The monosaccharide composition ratio of pectin was analyzed. Values are means ± S.D., n = 3. Asterisks represent statistically significant differences (* p<0.05, ** p< 0.01), as determined by student's t test. (C) The content of individual monosaccharides was determined. Values are means ± S.D., n = 3. Asterisks represent statistically significant differences (* p<0.05, ** p< 0.01), as determined by student's t test.

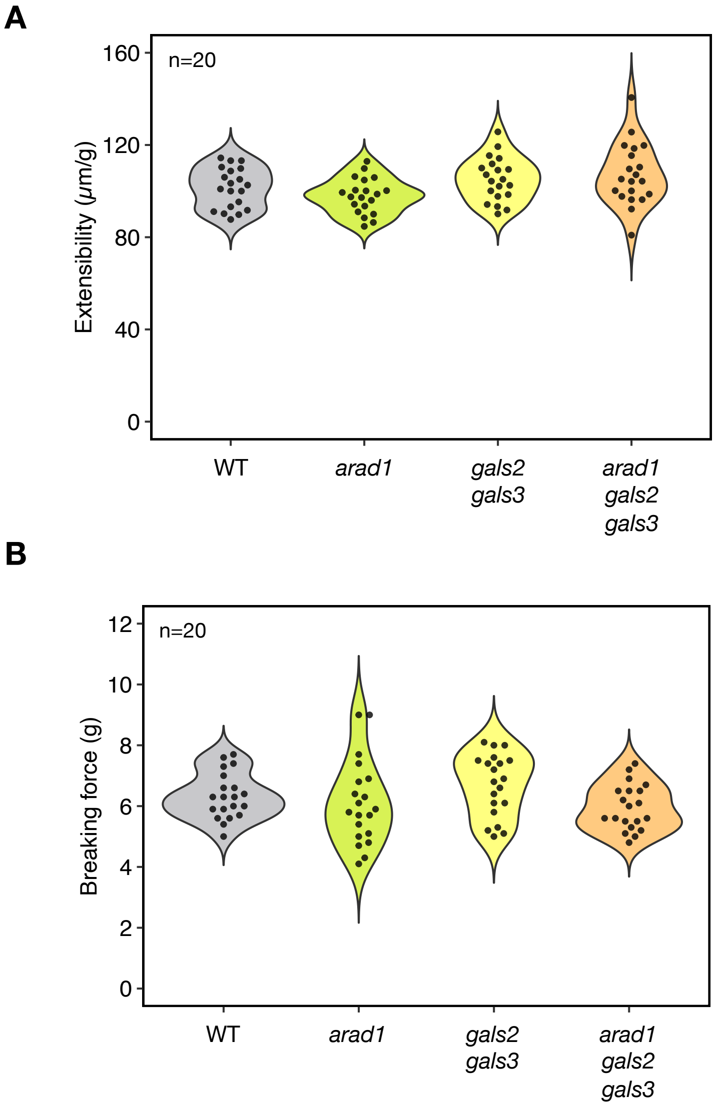

Fig. S2. Cell wall extensibility and breaking force of the rosette leaves of WT and *arad1 gals2 gals3*. (A) The cell wall extensibility of the rosette leaves from three-week-old WT, *arad1*, *gals2 gals3*, and *arad1 gals2 gals3* plants was measured, n = 20. (B) The breaking force of the rosette leaves from three-week-old WT, *arad1*, *gals2 gals3*, and *arad1 gals2 gals3* plants was evaluated, n = 20. No statistically significant differences were found in comparison with WT.

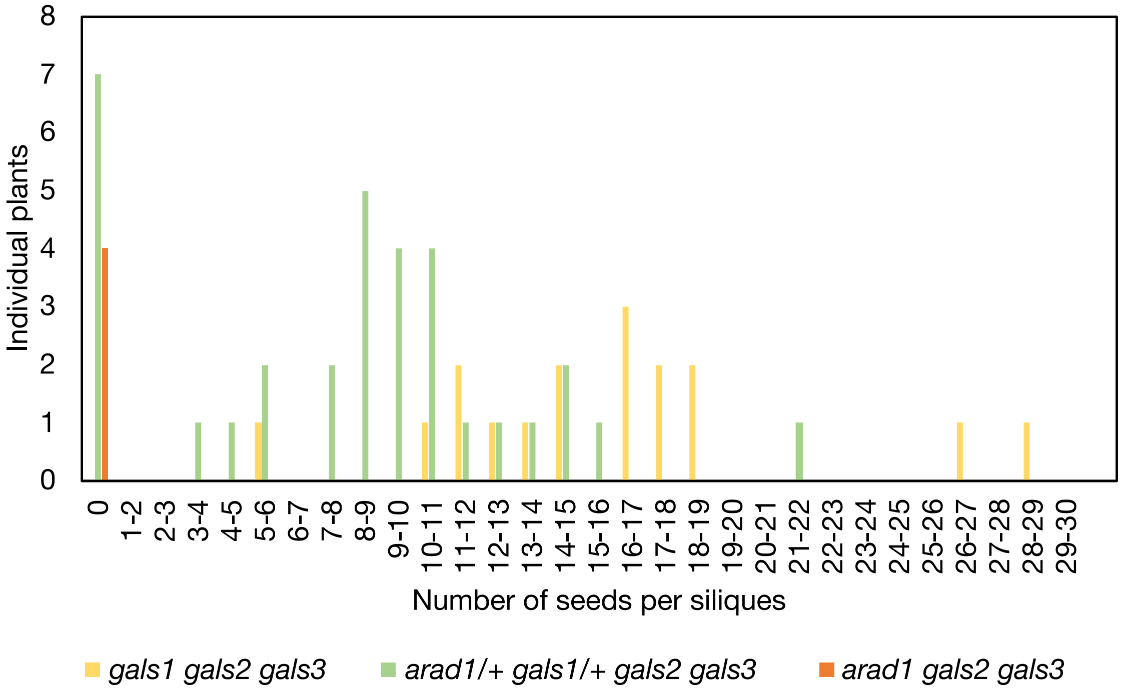

Fig. S3. Histogram of the number of seeds per silique in *gals1 gals2 gals3*, *arad1/+ gals1/+ gals2 gals3*, and *arad1 gals2 gals3*. The average number of seeds per silique was divided into bins and the number of individual plants in each bin is represented. Bars are color-coded for each silique genotype.

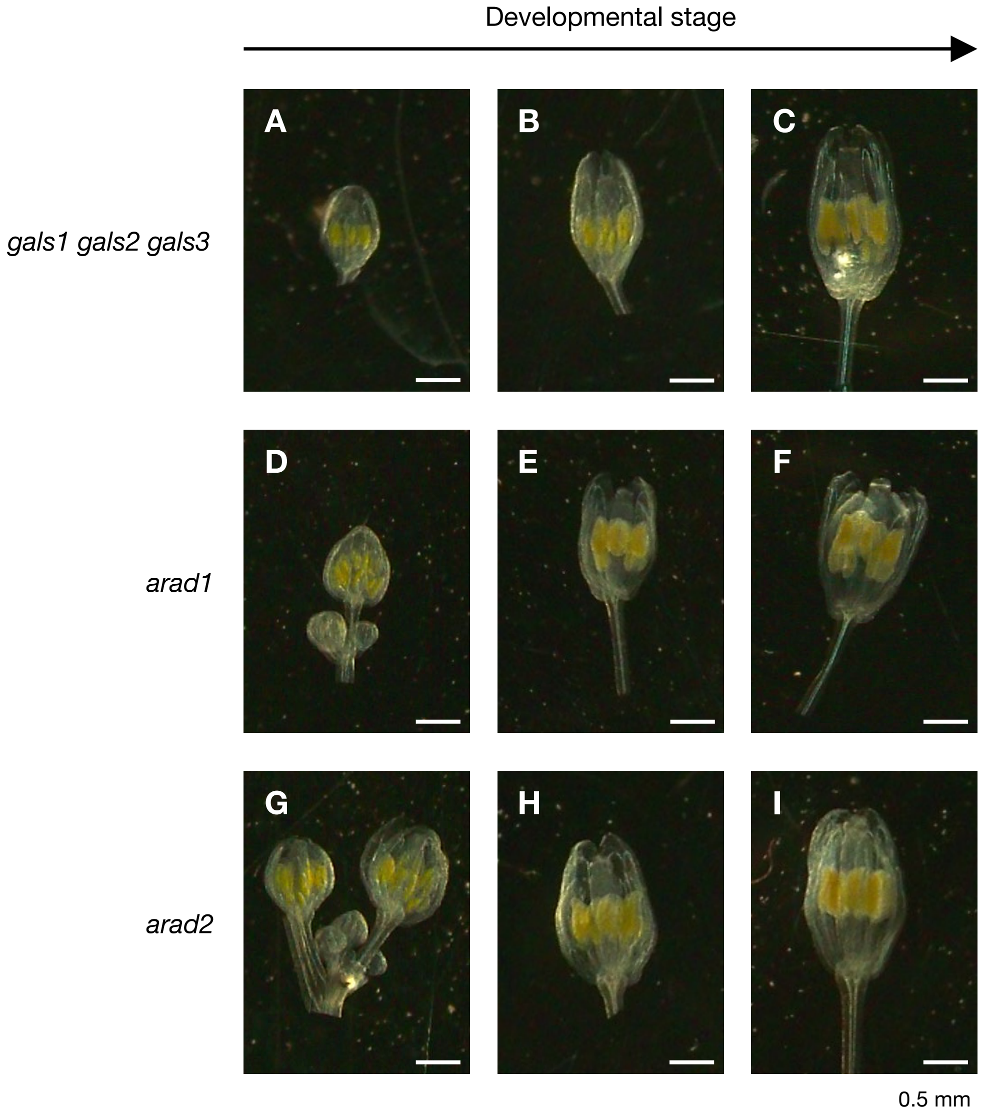

Fig. S4. Pollen inside buds was observed by making the tissue transparently visible. Stereomicroscopic images of pollen inside the buds of *gals1 gals2 gals3*, *arad1*, and *arad2* mutants. In all mutants, pollen was normally formed.

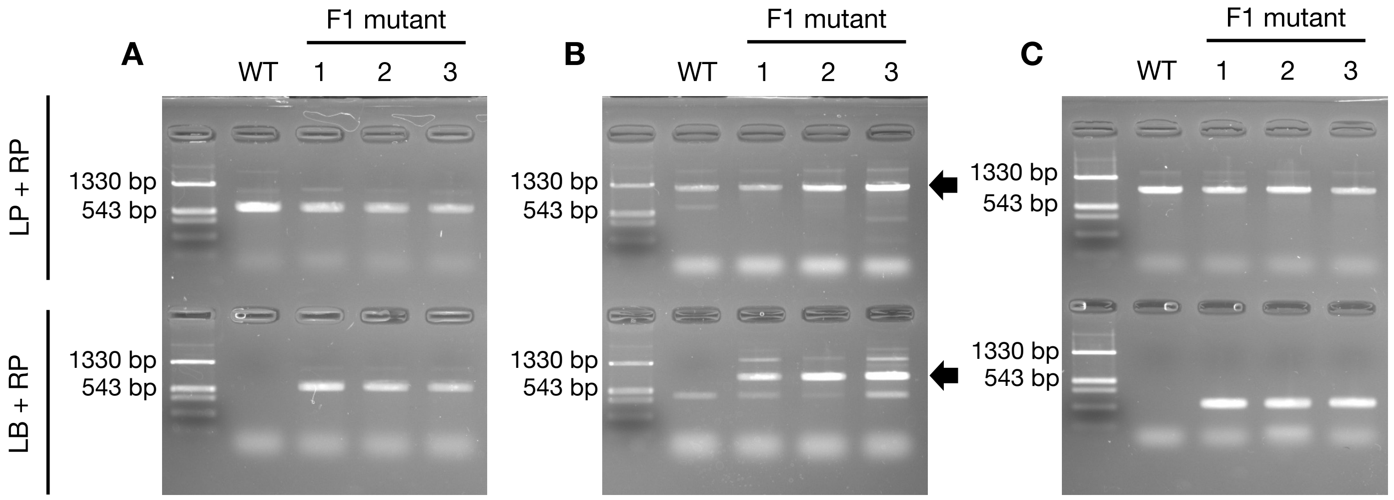

Fig. S5. The genotype of F1 seeds obtained by crossing WT pollen with *arad1 gals2 gals3* mutant. The genotype of F1 seeds obtained by crossing WT pollen with *arad1 gals2 gals3* mutant was analyzed using PCR. The result of performing PCR with *ARAD1* (A), *GALS2* (B) and *GALS3* (C) as the target gene. In each gel, the upper band represents the PCR performed with the left primer (LP) and right primer (RP) combination, while the lower band represents the PCR performed with the T-DNA left border primer (LB) and the RP combination. The arrows indicate the bands where the target gene was amplified.

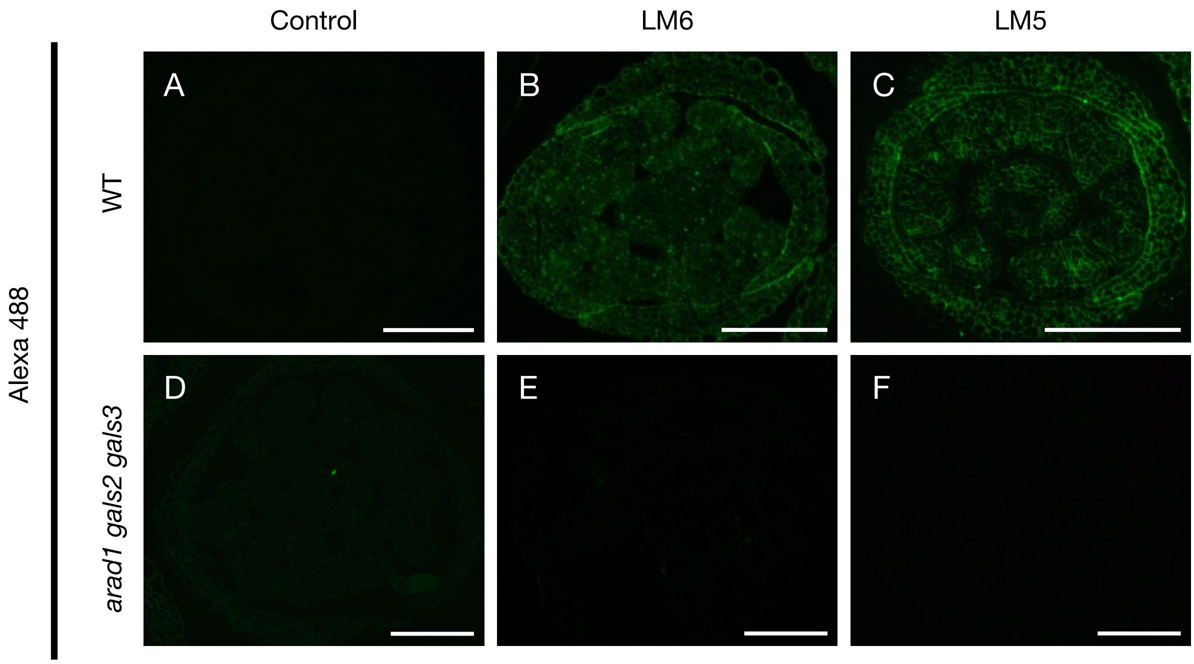

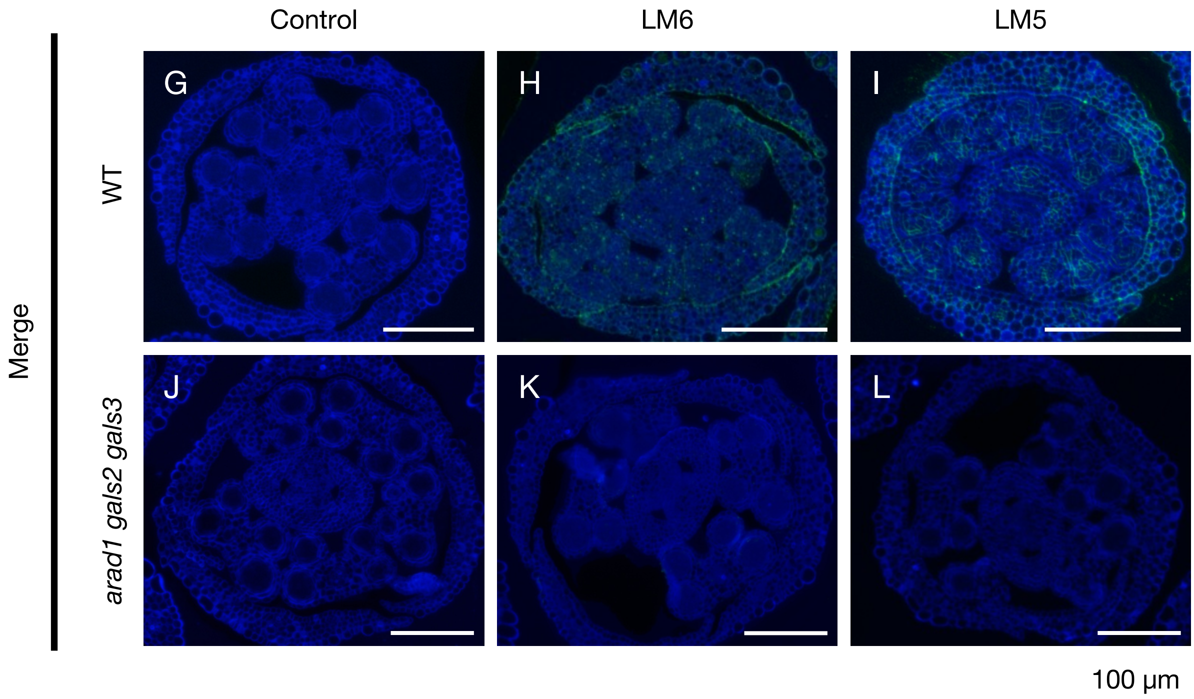

Fig. S6. Accumulation of arabinan and galactan in WT and *arad1 gals2 gals3* anther at stage 4. Observation of arabinan and galactan distribution in cross sections of an entire flower bud during anther and pollen development at stage 4 in WT and *arad1 gals2 gals3*. Scale bar = 100 µm. Control samples were prepared without using primary antibodies such as LM6 or LM5; only Alexa Fluor 488 and calcofluor white were used (A, D, G, J). Green-stained regions indicate arabinan and galactan accumulation detected with LM6 and LM5 respectively, and Alexa fluor 488 antibodies (B, C, E, F, H, I, K, L), and blue-stained regions indicate call wall accumulation with calcofluor white (H, I, K, L).

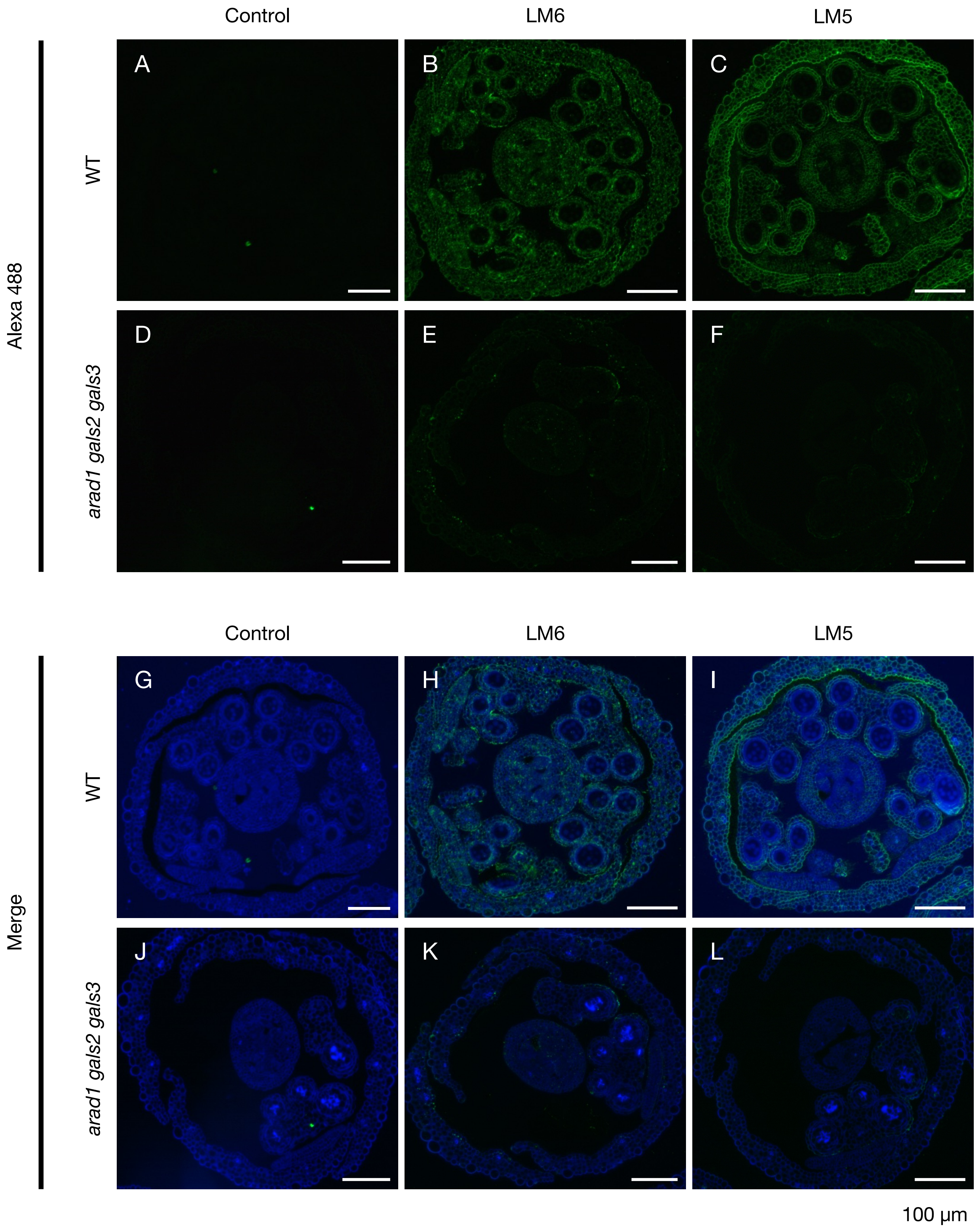

Fig. S7. Accumulation of arabinan and galactan in WT and *arad1 gals2 gals3* anther at stage 10. Observation of arabinan and galactan distribution in cross sections of an entire flower bud during anther and pollen development at stage 10 in WT and *arad1 gals2 gals3*. Scale bar = 100 μm. Control samples were prepared without using primary antibodies such as LM6 or LM5; only Alexa Fluor 488 and calcofluor white were used (A, D, G, J). Green-stained regions indicate arabinan and galactan accumulation detected with LM6 and LM5 respectively, and Alexa fluor 488 antibodies (B, C, E, F, H, I, K, L), and blue-stained regions indicate call wall accumulation with calcofluor white (H, I, K, L).

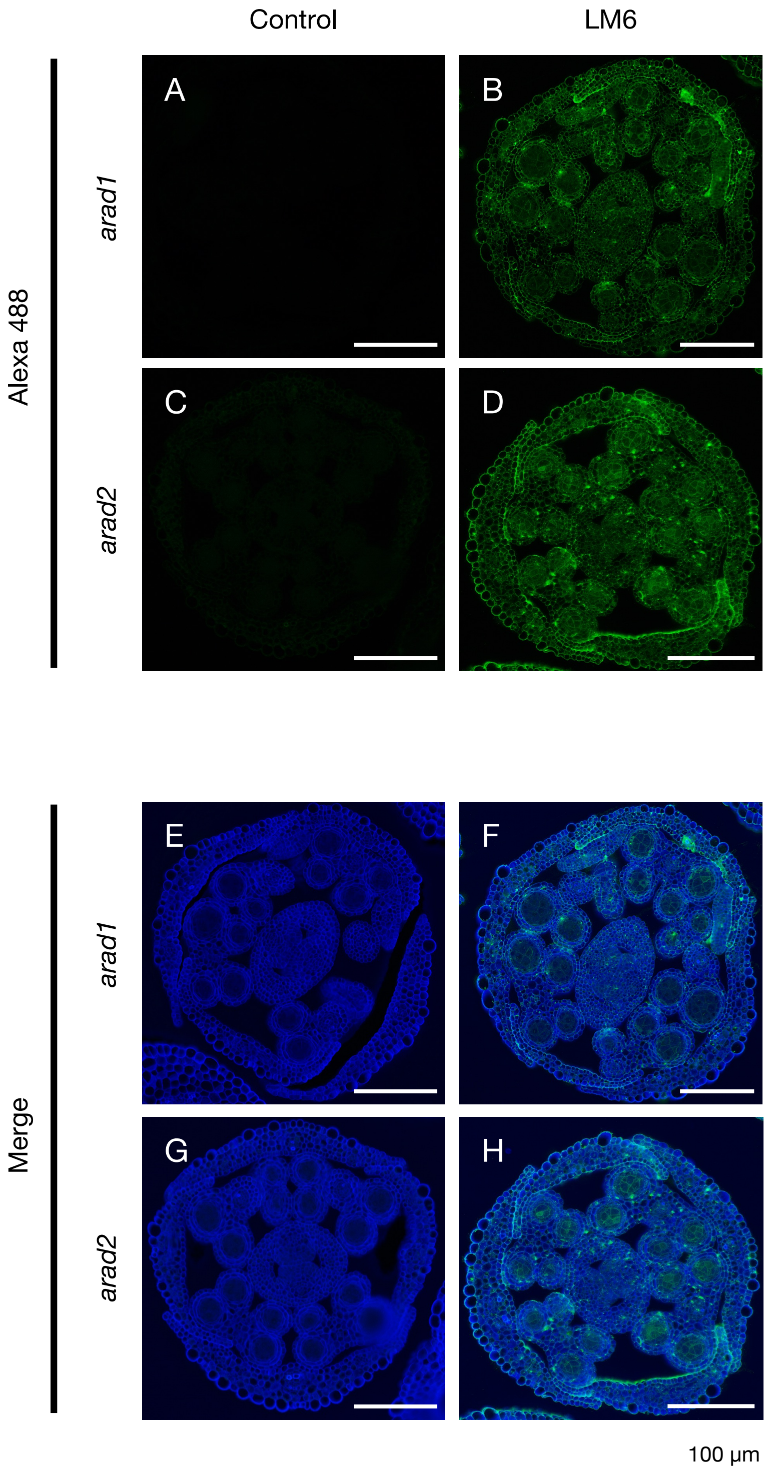

Fig. S8. Accumulation of arabinan in *arad1* and *arad2* anther at stage 5. Observation of arabinan distribution in cross sections of an entire flower bud during anther and pollen development at stage 5 in *arad1* and *arad2*. Scale bar = 100 μm. Control samples were prepared without using primary antibodies such as LM6; only Alexa Fluor 488 and calcofluor white were used (A, C, E, G). Green-stained regions indicate arabinan accumulation detected with LM6, and Alexa fluor 488 antibodies (B, D), and blue-stained regions indicate call wall accumulation with calcofluor white (F, H).

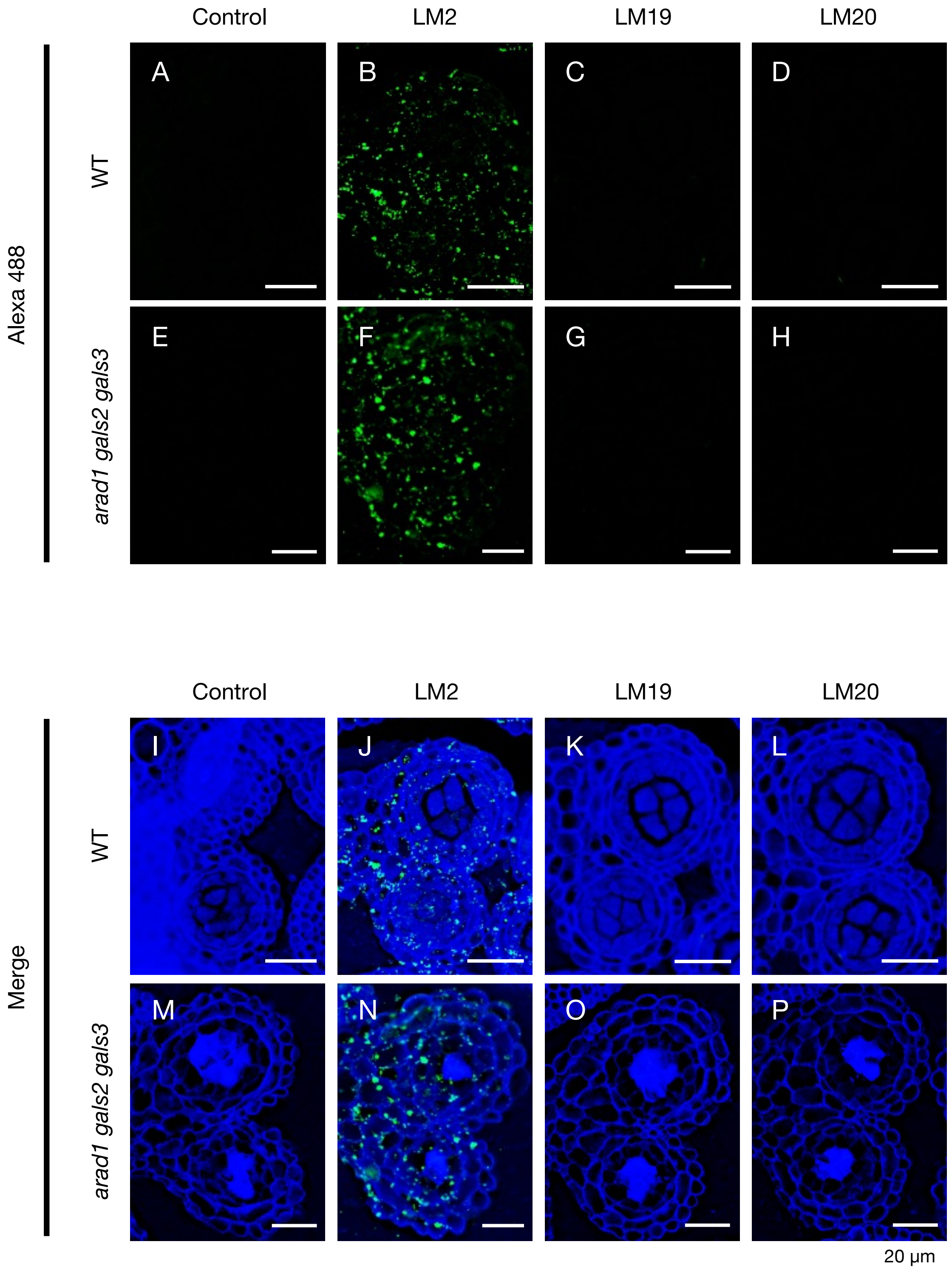

Fig. S9. Accumulation of AGP, un-methyl-esterified HG and methyl-esterified HG in WT and *arad1 gals2 gals3* anther at stage 5. Observation of AGP, un-methyl-esterified HG and methyl-esterified HG distribution in cross sections of PMCs at stage 5 in WT and stage 7 in *arad1 gals2 gals3*. Scale bar = 20 µm. Control samples were prepared without using primary antibodies only Alexa Fluor 488 and calcofluor white were used (A, E, I, M). Green-stained regions indicate AGP, un-methyl-esterified HG and methyl-esterified HG accumulation detected with LM2, LM19, LM20 respectively, and Alexa fluor 488 antibodies (B-D, F-H, J-L, N-P), and blue-stained regions indicate call wall accumulation with calcofluor white (J-L, N-P).

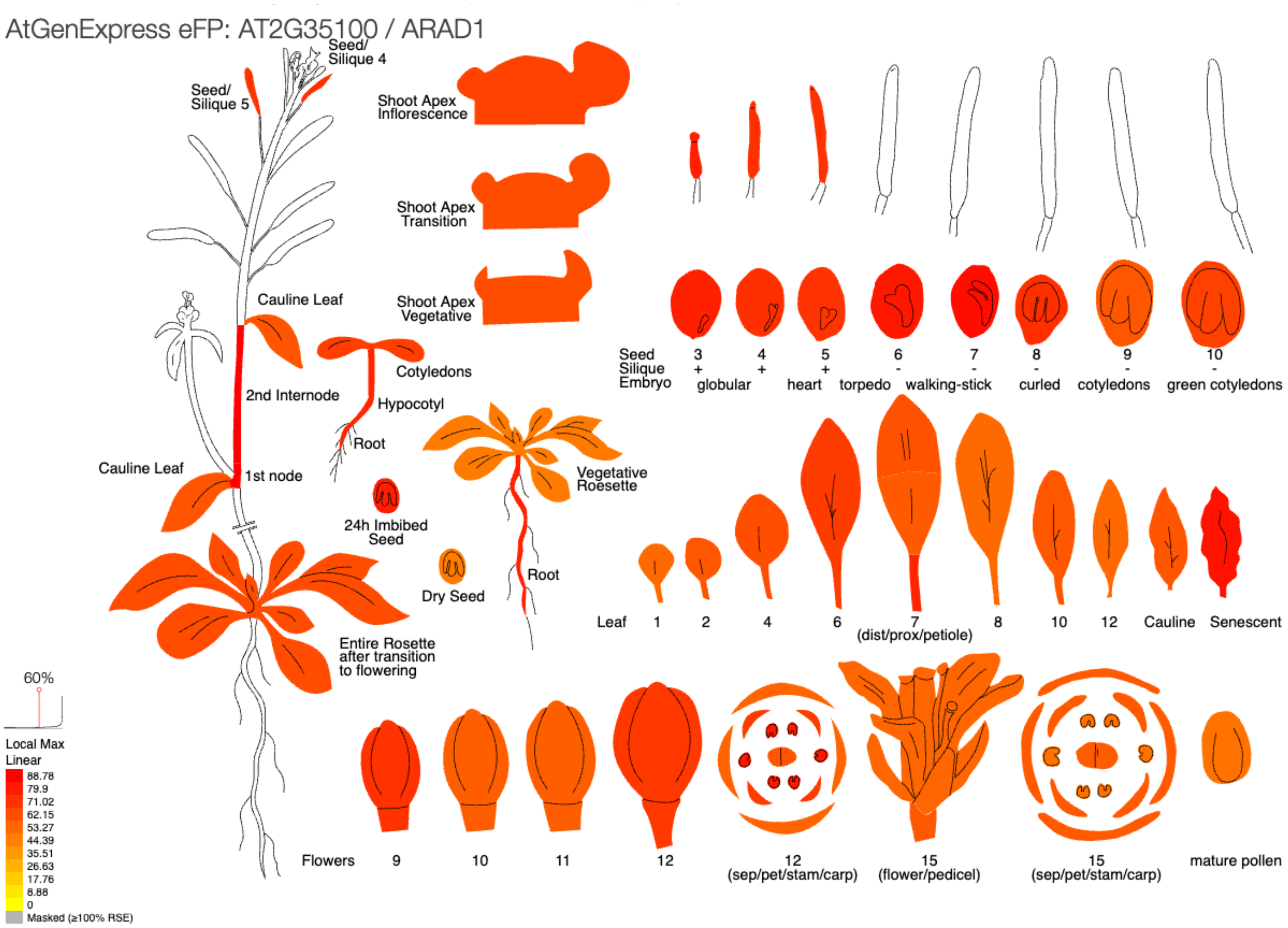

Fig. S10. Expression atlas of the Arabidopsis *ARAD1* gene across various tissues, retrieved from the eFP Browser.

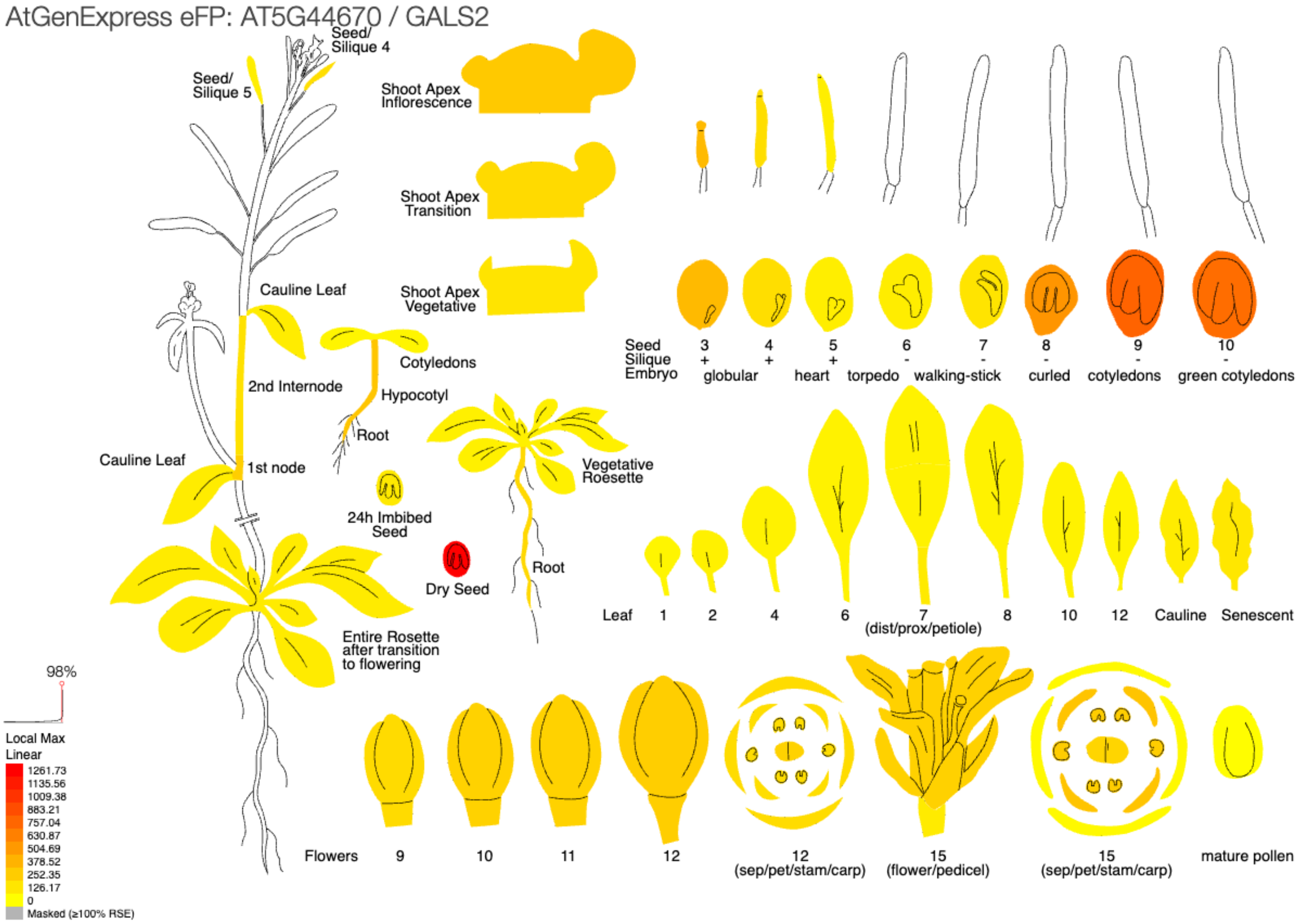

Fig. S11. Expression atlas of the Arabidopsis *GALS2* gene across various tissues, retrieved from the eFP Browser.

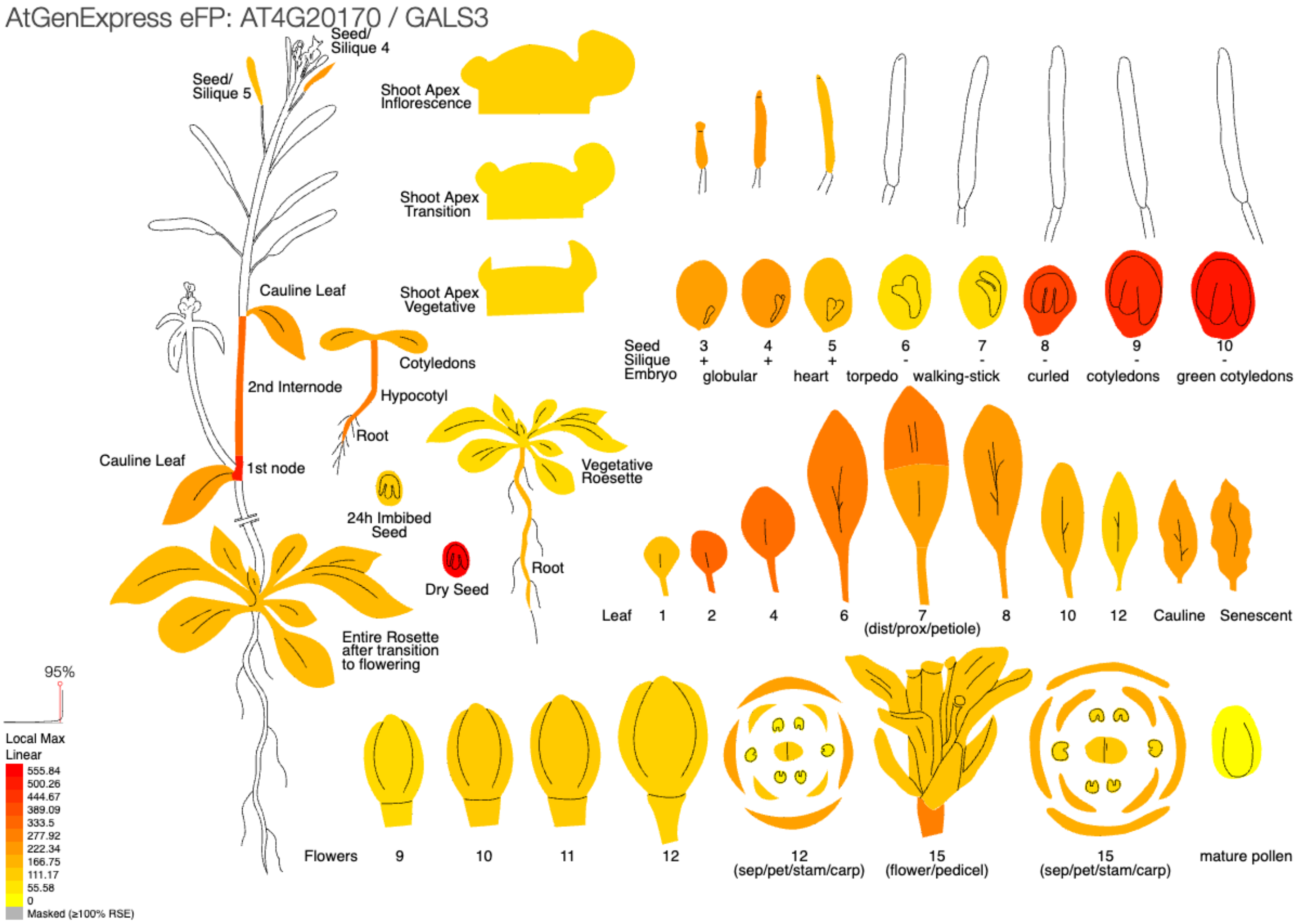

Fig. S12. Expression atlas of the Arabidopsis *GALS3* gene across various tissues, retrieved from the eFP Browser.

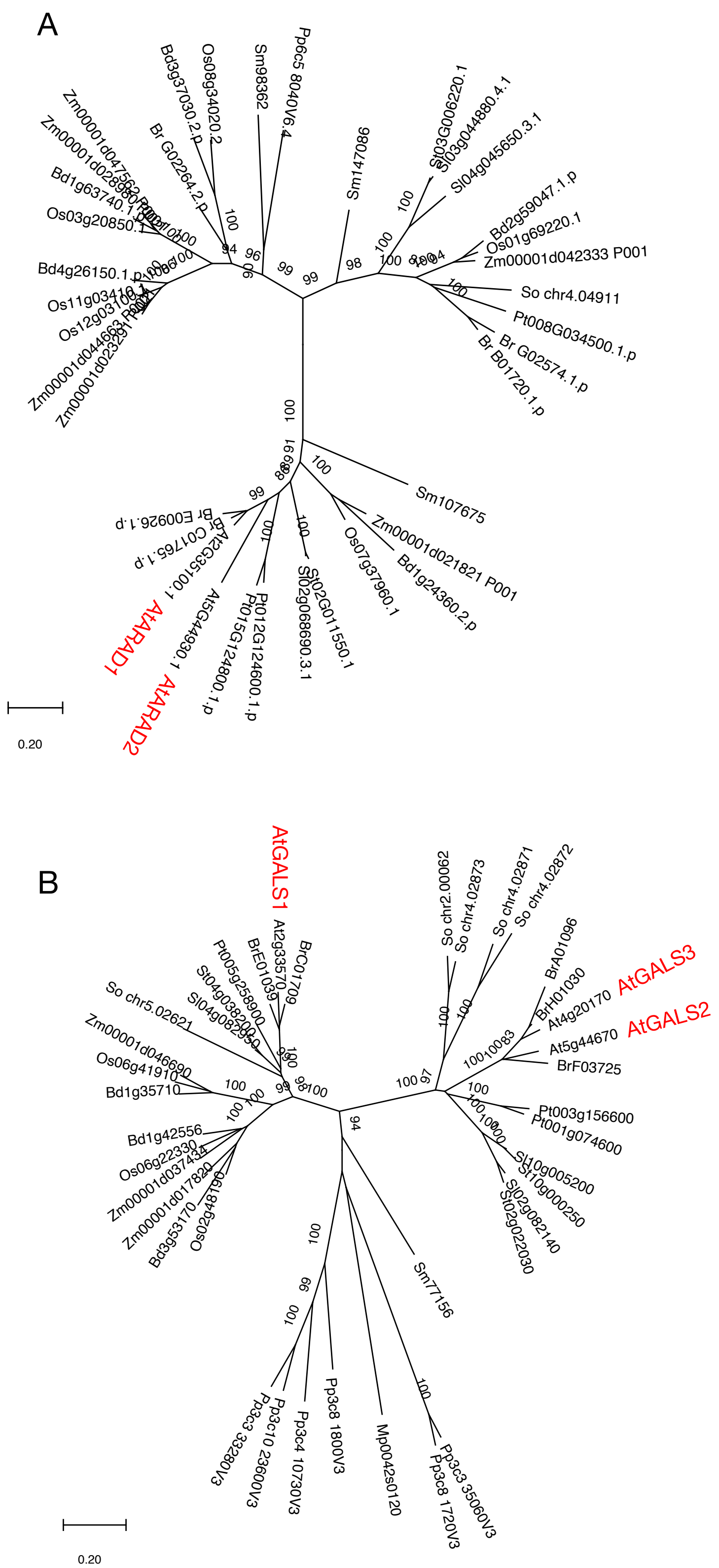

Fig. S13. Phylogenetic tree analysis of ARADs and GALSs. Amino acid sequences of various ARAD (A) and GALS (B) homologue proteins were obtained from Phytozome. Only bootstrap values higher than 80% are shown. At, *Arabidopsis thaliana*; Bd, *Brachypodium distachyon*; Br, *Brassica rapa*; Os, *Oryza sativa*; Pp, *Physcomitrium patens*; Pt, *Poplus trichocarpa*; Sl, *Solanum lycopersicum*; Sm, *Selaginella moellendorffii*; So, *Spinacia oleracea*; St, *Solanum tuberosum*; Zm, *Zea mays*.
